# Genome Analysis Using Whole-Exome Sequencing of Non-Syndromic Cleft Lip and/or Palate from Malagasy Trios Identifies Variants Associated with Cilium-Related Pathways and Asian Genetic Ancestry

**DOI:** 10.3390/genes14030665

**Published:** 2023-03-07

**Authors:** Zarko Manojlovic, Allyn Auslander, Yuxin Jin, Ryan J. Schmidt, Yili Xu, Sharon Chang, Ruocen Song, Sue A. Ingles, Alana Nunes, KC Vavra, Devin Feigelson, Sylvia Rakotoarison, Melissa DiBona, Kathy Magee, Operation Smile, Anjaramamy Ramamonjisoa, William Magee III

**Affiliations:** 1 Department of Urology, Keck School of Medicine of USC, Los Angeles, CA 90033, USA; 2 Department of Translational Genomics, Keck School of Medicine of USC, Los Angeles, CA 90033, USA; 3 Operation Smile Inc., Virginia Beach, VA 23453, USA; 4 Division of Plastic and Maxillofacial Surgery, Children’s Hospital Los Angeles, Los Angeles, CA 90027, USA; 5 Department of Pathology and Laboratory Medicine, Children’s Hospital Los Angeles, Los Angeles, CA 90027, USA; 6 Department of Pathology, Keck School of Medicine of USC, Los Angeles, CA 90033, USA; 7 Department of Population and Public Health Sciences, Keck School of Medicine of USC, Los Angeles, CA 90033, USA; 8 Agilent Technologies, Inc., Santa Clara, CA 95051, USA; 9 Operation Smile Madagascar, Antananarivo 105, Madagascar; 10 Division of Plastic and Reconstructive Surgery, Keck School of Medicine of USC, Los Angeles, CA 90033, USA

**Keywords:** cleft lip, cleft palate, whole-exome sequencing, genetic ancestry, primary cilia, genetic syndrome

## Abstract

Background: Orofacial clefts (OFCs) are common congenital disabilities that can occur as isolated non-syndromic events or as part of Mendelian syndromes. OFC risk factors vary due to differences in regional environmental exposures, genetic variants, and ethnicities. In recent years, significant progress has been made in understanding OFCs, due to advances in sequencing and genotyping technologies. Despite these advances, very little is known about the genetic interplay in the Malagasy population. Methods: Here, we performed high-resolution whole-exome sequencing (WES) on non-syndromic cleft lip with or without palate (nCL/P) trios in the Malagasy population (78 individuals from 26 families (trios)). To integrate the impact of genetic ancestry admixture, we computed both global and local ancestries. Results: Participants demonstrated a high percentage of both African and Asian admixture. We identified damaging variants in primary cilium-mediated pathway genes *WNT5B* (one family), *GPC4* (one family), co-occurrence in *MSX1* (five families), *WDR11* (one family), and tubulin stabilizer *SEPTIN9* (one family). Furthermore, we identified an autosomal homozygous damaging variant in *PHGDH* (one family) gene that may impact metabiotic activity. Lastly, all variants were predicted to reside on local Asian genetic ancestry admixed alleles. Conclusion: Our results from examining the Malagasy genome provide limited support for the hypothesis that germline variants in primary cilia may be risk factors for nCL/P, and outline the importance of integrating local ancestry components better to understand the multi-ethnic impact on nCL/P.

## 1. Introduction

Orofacial clefts (OFCs) are among the most common birth defects worldwide [1]. The incidence rate is approximately 1 in 700 live births globally, but varies widely by ethnicity, gender, and cleft phenotype [1,2]. The highest rates of OFCs are found in Asian populations, with an incidence as high as 1 in 500 live births [3]. The lowest rates are found in African populations (1 in 2000 live births), with populations of European ancestry falling in the middle at approximately 1 in 1000 live births [1,4,5]. Patients with OFCs are considered non-syndromic in the absence of any other birth defect, which accounts for approximately 70% of cases of cleft lip with or without palate (CL/P) and 50% of cases of isolated cleft palate (iCP) [1,6,7]. Although the causes of syndromic clefts are considered mainly genetic, the etiology of non-syndromic cleft lip with or without cleft palate (nCL/P) remains unclear.

There have been 14 major genome-wide association studies (GWAS) that have reported associations between common genetic variants and OFCs [8,9,10,11,12,13,14,15,16,17,18,19,20,21]. A recent meta-analysis assessed 31 case–control studies focused on nCL/P with respect to *IRF6* or 8q24, the two main regions consistently associated with this disease [20]. This paper highlights a considerable heterogeneity within the findings by race/ethnicity—specifically between those of Asian and African descent. This is continually reflected by the literature surrounding all known risk loci, as there is a lack of understanding of the attributable percentage of disease risk they account for by race/ethnicity. A recent GWAS in an entirely African cohort found that although 8q24 is still the most significant locus for nCL/P (consistent with other studies), the most significant SNP they found was not the same as has been reported among Europeans [21]. Although many studies have been successful in identifying risk loci associated with a cleft, the diversity in the populations is still minimal and does not include many of the individuals at the highest risk of living with the disease.

Whole-exome sequencing (WES) has been utilized to identify pathogenic single nucleotide polymorphisms (SNPs) along with small insertions and deletions correlated with nCL/P in European, Asian, South American, and Arab populations [22,23,24,25,26,27]. Specifically, regions of interest have been identified in individuals of Indian [24], German [24], European-American [22,23,24], Filipino [22,23,24], Syrian [24], Honduran [24], Chilean [22], Danish [22], Uruguayan [22], Japanese [22], Vietnamese [22], and Chinese descent [25,26,27]. To our knowledge, African populations have not been represented.

While efforts are being made to include traditionally excluded populations in genetic research, many populations are still under-represented in the literature. Over 75% of genetic research studies have been performed on individuals of European ancestry, even though they represent less than 25% of the global population [28]. Additionally, it has been found that 78% of all GWAS studies are of European ancestry, with only 2% African representation and 8% East Asian representation [28]. Only 19 genetic-focused studies have been conducted that included Malagasy populations [29,30,31,32,33,34,35,36,37,38,39,40,41,42,43,44,45]. Most of these studies utilize genetic techniques to investigate the genetic lineages of Malagasy populations, whose ancestral history has been widely disputed. It is believed to be a unique combination of African and Asian (specifically Bantu and Austronesian) ancestry; however, these studies only speak generally to the human history of Madagascar [29,34,35,36,39,42,45], with only one study looking at prevalence of oral clefts in Madagascar [46]. Lastly, Bloch-Zupan et.al., in their study, performed WES in a small region in Madagascar and identified a novel single nucleotide deletion in the *DSPP* gene confirming a clinical diagnosis of dentinogenesis imperfecta. However, they did not assess the ancestry of their participants more widely than a five-generation familial pedigree and failed to identify the ancestral population origins that could contribute to inheritance of this trait [32]. The global body of knowledge about Malagasy populations generally, their genetic ancestry, and how this impacts population health in Madagascar is minimal. As it is one of the poorest countries globally, it is an important target population for inclusion in the literature to best serve patients who are the least likely to have access to care.

Molecular drivers of cleft lip with or without palate remain elusive and complex, with recent evidence implicating primary cilia as a potential candidate [47]. Primary cilia are evolutionarily conserved microtubule-based structures on the surface of epithelial and most other cells that have emerged as critical regulators of developmental signaling pathways [48]. These types of cilia also play a crucial role in embryonic development, which is essential for cell polarity and neural tube development [49,50]. Furthermore, these cilium structures are vital regulators of signaling pathways critical for normal craniofacial morphogenesis, such as Wnt, Hedgehog, fibroblast- and platelet-derived growth factors [51]. Although ciliopathies have been identified as an important factor in normal craniofacial development [47], nothing is known about variants in cilium-associated genes in the Malagasy population.

In the current study, we aim to identify disease-causing genetic variants of nCL/P in 26 case–parent trios (*n* = 78) in a population from Madagascar with no family history of OFC, using WES. This study analyzes WES data in four ways: single-variant analysis, gene-based analysis, ancestry analysis, and copy-number-variant analysis. Secondarily, as there is a lack of Malagasy population representation within the global Genome Project [52], we plan to add to the geographic human genome map. Collectively, these findings will aid in the genetic understanding of the Malagasy people and population-specific risk loci that may contribute to the risk of nCL/P. Identifying additional genetic factors contributing to nCL/P, especially in populations traditionally missing from the literature, could aid in improving diagnostics, treatment, and outcomes for these patients.

## 2. Results

### 2.1. Study Outline

This study was conducted on 26 case–parent trios from Madagascar at the Centre Hospitalier de Référence Régional (CHRR), the regional referral hospital in Antsirabe (Table 1). The cohort was well-balanced for gender at 50%/50% (*n* = 13 per group), with the major clinical phenotype being unilateral cleft lip and palate at 62% (*n* = 16), followed by isolated cleft lip at 31% (*n* = 8), and bilateral cleft lip and palate at 8% (*n* = 2). Whole-exome sequencing utilized for the downstream analysis was performed, and we archived a mean 172.1 depth coverage with target bases greater than 10× at 95% (Table 1, Supplementary Table S1).

### 2.2. Characterization of Madagascar Trios by Genetic Ancestry

**Figure 1:**
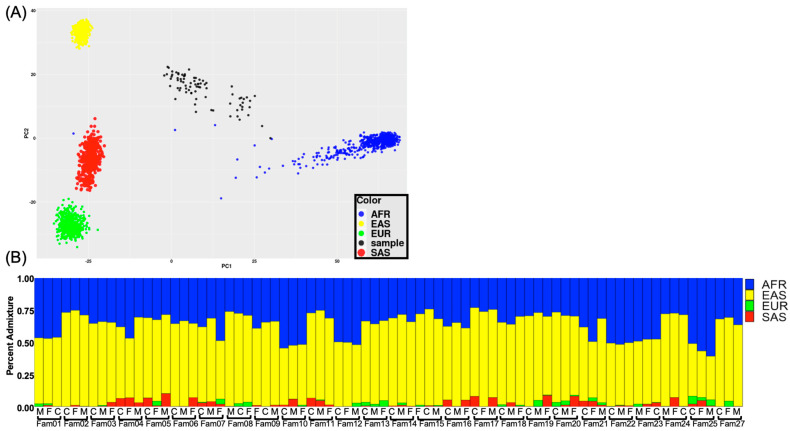
Population stratification defines propositions of calculated genetic ancestry. (**A**) Principal component analysis (PCA) across all subjects in this study (black dots). The European ancestry (green dots), African ancestry (blue dots), East Asian ancestry (yellow dots), and South Asian ancestry (red dots) using AIMs derived from the custom whole-exome deep sequencing and matched to 1k Genome Population Exome Phase1_v3 Genotypes. (**B**) STRUCTURE plot (K = 4; 50,000 Burn-in period and 100,000 NUMREPS) used to compute genetic ancestry percent admixture: European ancestry (green), African ancestry (blue), South Asian ancestry (red), and East Asian ancestry (yellow). Each trio family cluster contains M (mother), F (father), and C (child).

To compute genetic ancestry, we performed principal component analysis (PCA) to cluster participants by genetic ancestry informative markers (Figure 1A). Furthermore, to assess the percent ancestry admixture, we performed STRUCTURE (Figure 1B). Both analyses indicated an admixture of African (mean = 37%) with enrichment of East Asian (mean = 60%) ancestries among participants (Figure 1B). Lastly, to assess local ancestry from each case, we performed Local ancestry in admixed populations (LAMP) (Supplementary Figure S2). Since incidents of nCL/P are observed at the highest rates in the Asian population with the lowest in African populations [1,5,6], we performed a Chi-square analysis across the entire genome between two cohorts (children (case) vs. parents (family-based control)) to assess the unsupervised local ancestry significance of Asian homozygosity in this admixed population. The Chi-square analysis identified significant regions (*p* < 0.05) of genome-wide East Asian homozygosity haplotypes that encompassed genes associated with nCL/P, among others (Supplementary Table S2).

### 2.3. Variant Analysis Identifies Genes That are Associated with Ciliopathies

Variant calling and inheritance annotation were performed by the Alissa Interpret (Agilent) software package using children as cases and parents as a family-based control group. Each annotated missense variant effect was checked across multiple prediction packages to assess its pathogenic likelihood.

**Figure 2:**
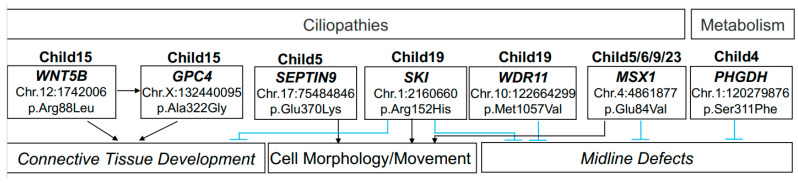
Diagram of variants outlined in our study. The pathways were assessed by KEGG and Ingenuity Pathway Analysis (IPA). The top box indicates a major function of these genes; the middle panel indicates the pedigree and a description of the variant. The bottom panels indicate major disease or function annotation with black errors, indicating the direction of activation and blue lines indicating inhibition within the network.

In Family 15, the child with bilateral cleft lip and palate carries a rare missense (p.Arg88Leu) heterozygous nucleotide variant (c.263G > T) in exon 3 (rs200966877) inherited from the father on chromosome 12 of WNT family member 5B (*WNT5B*) gene (Figure 2, Table 2, Supplementary Table S6). The coverage of the *WNT5B* c.263G > T variant is 123x and located in the Disulfide Bond region (predicted by UniProt) that may be deleterious by LRT prediction and is identified as disease-causing by mutation taster predictor (Table 2, Supplementary Table S6). The variant is also predicted to be damaging by PROVEAN and SIFT (SIFT = 0.025). The T allele in *WNT5B* at Chr.12: 1742006 is rare at a frequency of 1/246,220 in the general population reported in gnomAD (alleleFrequencyAll) (Table 2, Supplementary Table S6). The local ancestry reveals that the variant is in an East Asian haplotype (Supplementary Figure S2; Family_015). The child (Family 15) also carries a variant in glypican-4 (*GPC4*) that is associated with the regulatory role of Wnt pathways and the activity of β-catenin signaling [53]. The heterozygous missense *GPC4* variant (p.Ala322Gly) is inherited from the mother and is located on the X-chromosome: 132440095 (c.965C > G). The alternate G allele position is sequenced at a coverage of 169x and is not reported in the general population by gnomAD. The variant is predicted to be deleterious by LRT, disease-causing by Mutation Taster, and damaging by PROVEAN and SIFT (Table 2, Supplementary Table S6).

The child in Family 19 with unilateral cleft lip and palate has two variants in genes associated with ciliopathies. One is in the gene *SKI* located on chr.1: 2160660 (c.455G > A) (Figure 2, Table 2, Supplementary Table S6). The nonsynonymous variant results in p.Arg152His change on exon 1 has not been reported in the general population (Table 2, Supplementary Table S6). The amino acid change is located between two helix structural protein features predicted by UniProt. Furthermore, it is predicted to be deleterious by LRT, disease-causing by Mutation Taster, and damaging by PROVEAN and SIFT (Table 2). The gene *SKI* is located on a heterozygous African–East Asian local ancestry haplotype (Supplementary Figure S2; Family_019). The other variant in the WD repeat domain 11 (*WDR11*) gene is inherited from the mother by heterozygous mode located on chr.10: 122664299 (c.3169A > G) (Figure 2, Table 2, Supplementary Table S6). The variant results in p.Met1057Val show an amino acid change, which has not been reported in the general population and is predicted to be damaging by LRT, SIFT, and PPH2 as well as neutral by PROVEAN (Table 2, Supplementary Table S6). *WDR11* is predicted to be in heterozygous Asian and African ancestry loci.

The variant c.1108G > A in gene *SEPTIN9* (rs1297513860) was inherited from the mother by heterozygous mode in child 5 with unilateral cleft lip (Figure 2, Table 2). The variant is located on chr.17: 75484846 and has 1/246,242 allele frequency in the general population by gnomAD (Table 2, Supplementary Table S6). The missense p.Glu370Lys variant is predicted to be disease-causing and damaging by in silico mutation assessor tools (Table 2, Supplementary Table S6). The *SEPTIN9* gene resides in the homozygous Asian locus in the proband (Supplementary Figure S2; Family_005).

In child 4 with unilateral cleft lip and palate, we identified an autosomal homozygous pattern of inheritance in the phosphoglycerate dehydrogenase (*PHGDH*) gene (rs143340742) located on chr.1: 120279876 (c.932C > T). The T allele is present at a frequency of 2/276,962 in the gnomAD general population. The missense variant *PHGDH* p.Ser311Phe is predicted to be neutral by LRT, low by Mutation Assessor, damaging, and disease-causing by Mutation Taster, SIFT, PPH2, and PROVEAN. *PHGDH* gene is located on the homozygous locus for East Asian ancestry in the proband but is heterozygous for South Asian and African in parents, suggesting that the East Asian gene was inherited (Supplemental Figure S2; Family_004).

Lastly, we detected a recurrent nonsynonymous variant in the muscle segment homeobox 1 (*MSX1*) gene (rs28928890) inherited from the mother as a heterozygous candidate (Figure 2, Table 2). The missense variant is present in four male cases with high coverage ranging between 66 and 89x: proband 5 with unilateral cleft lip, proband 6 with unilateral cleft lip and palate, proband 9 with unilateral cleft lip and palate, and proband 23 with unilateral cleft lip (Figure 2, Table 2). The variant is located on chr.4: 4861877 (c.251A > T) with the missense mutation change of p.Glu84Val. The variant was initially reported in ClinVar as pathogenic in Orofacial cleft 5 [22] (ID = 14883) but has changed to uncertain significance in the recent 2022 submission (Table 2). The alternative T allele has a population frequency of 1/102,294 in genomAD. The variant did not show significance in family-based genome-wide association analysis in our modest sample size (*n* = 26 trios). Further analysis of the *MSX1* percent local ancestry also did not show significant ancestry-related enrichment, but all cases have at least one East Asian allele in that locus (Supplementary Figure S2).

### 2.4. Copy-Number Analysis Reveals CNVs in Genes Associated with Metabolism and Ciliopathies

Here, we performed a copy-number-variant (CNV) analysis on high-resolution WES (additional probes evenly distributed across the entire genome for improved CNV calling) to identify candidate structural variants in nCL/P families from Madagascar. We performed extensive quality controls (methods) to ensure only high-quality CNV calling in probands after subtraction for family-based controls, geographic/ancestry-related components, and relative technical components. We observed high-quality data with a QC mean score of 0.019 and observed an average of 97 CNVs per individual (min = 72 CNVs; max = 217 CNVs) (Supplementary Table S3). We did not observe a significant difference in CNVs between proband and family control samples. Additionally, focal analysis using the GISTIC2.0 approach identified 47 significant events in children (Q-bound of <0.001 (FDR)) with nCL/P (Supplementary Figure S4, Supplementary Table S4) that were also present in parent controls. This suggests that these may be population-specific events not associated with pathogenesis (Supplementary Figure S4, Supplementary Table S4).

**Figure 3:**
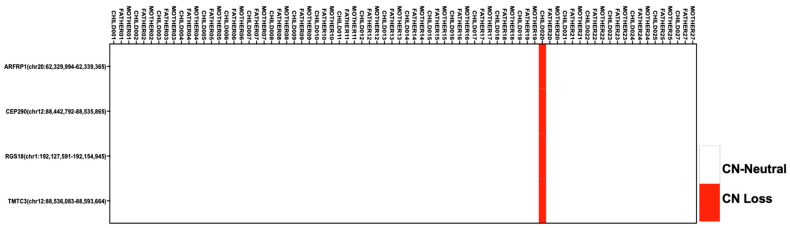
Copy-number variants that are unique to the proband and not present in the general population and control parents. Gene and location are shown for each copy-number event. The white indicates copy neutrality, and the red indicates copy loss. Each CNV is mapped across all trios.

As the modest study size might be underpowered to detect statistically significant events, we performed a manual review to identify de novo CNVs that were only present in cases and not in any of the controls. Through this process, we identified four copy losses in affected child 20, male with unilateral cleft lip and palate (Figure 3). All four CNV regions were detected as copy loss events and were located on chr1:192, 127, 591–192, 154, and 945 in the Regulator of G-protein signaling 18 (*RGS18*) gene, chr12:88, 442, 792–88, 535, and 865 in the Centrosomal protein of the 290 kDa (*CEP290*) gene, chr12:88, 536, 083–88, 593, and 664 in the transmembrane O-mannosyltransferase targeting cadherins 3 (*TMTC3*) gene, and chr20:62, 329, 994–62, 339, and 365 of ADP-ribosylation factor-related protein 1 (*ARFRP1*) (Figure 3, Supplementary Table S5).

## 3. Discussion

nCL/P is a multifactorial disorder driven by combinatory genetic and environmental factors at the early stages of embryonic development, with large population differences in incidence rates. The geographical differences in incidents of nCL/P range from an average of ~1.48 clefts (all phenotypes) per 1000 live births in Asians, ~1.42 per 1000 in Hispanics, and ~1.00 per 1000 in non-Hispanic Whites, to ~0.89 per 1000 in populations of African descent [1,3,5,54]. To better understand geographical differences as a function of genetic ancestry and link them to the genetic risk factors associated with nCL/P, we performed our analysis in a Malagasy admixed population. To date, this is the first report of genetic work in Madagascar to identify disease-causing genetic variants from protein-expressing regions of the genome in nCL/P case–parent trios. Here, we have identified genes that may contribute to the risk of nCL/P influenced by the over-transmission of East Asian ancestry-enriched alleles.

We have identified multiple genetic alterations that, although present across different genes, share a biological link to cilia that suggests a potential class of craniofacial ciliopathies. In addition, all events are present on haplotypes associated with at least one East Asian allele, which may provide limited support for observed geographical differences in OFC incidents. The primary cilium is an important finger-like organelle present in most vertebrate cells that acts as a major pathway hub for multiple key developmental signaling pathways necessary during embryonic development as well as the general functionality of mature cells. Some of the major pathways regulated by cilia are WNT, platelet-derived growth factor, and Hedgehog signaling [47]. Due to such a central role in embryonic development, dysfunctions of structure or function in cilia are known as ciliopathy [47]. Ciliopathies are phenotypically variable, with the most common phenotype being craniofacial defects that range from midline defects to OFCs [47]. The most common clinical manifestations associated with craniofacial dysmorphologies are Bardet–Biedl, Joubert, Meckel–Gruber, orofaciodigital, and Ellis–van Creveld syndromes [47].

The predicted damaging variant in the *SKI* gene (c.455G > A) in affected child 19 may play a key role in inhibiting connective tissue development and events associated with midline defects linked to nCL/P [55]. The *SKI* protein is an inhibitor of TGF-β by interacting with the SMAD complex and preventing nuclear entry [56]. Furthermore, *SKI* has also been shown to act as a corepressor of Gli partner proteins that are localized in primary cilia and are critical regulators of the Hedgehog signaling pathway, which may be one possible passenger of the observed phenotype [57,58]. Child 19 also has a damaging inherited variant in the *WDR11* gene that is an integral part of the Hedgehog signaling pathway, with impairments leading to ciliopathies [59]. These two heterozygous hits potentially impair the same pathway and may suggest a possible link to nCL/P.

Mutations in *MSX1* are expressed in embryonic tissue and have been associated with nCL/P as well as other craniofacial syndromes [60]. Studies have linked variants in the *MSX1* gene to Asian [61,62] and African [63,64] populations, and including the damaging variant (rs28928890) in *MSX1* that is located on an East Asian/African admixed locus might suggest further evidence of the importance of integrating local ancestry in future studies to better understand the ancestral impact on this locus. This will further empower a better understanding of population contributions to intrinsic genetic structures.

Affected child 5, who has a variant in the *MSX1* gene, also inherited a *SEPTIN9* (rs1297513860) variant. This gene is critical for filament formation, cellular movement, and stability [65]. It is inherited through the mother by heterozygous mode. Interestingly, although both parents are heterozygous for African/East Asian local ancestry, the affected child is homozygous for East Asian ancestry. This may further provide evidence of the potential ancestorial impact on incidence rates in Asians with nCL/P, highlighting the importance of understanding local ancestry in complex populations.

*WNT5B* and *GPC4* are cilium-related genes that have been shown to regulate WNT pathway signaling and ciliogenesis [66,67,68]. Inherited damaging variants in *WNT5B* and *GPC4* in affected child 15 may play a role in the development of nCL/P through potential ciliopathic mode. The affected child 15 is homozygous for the East Asian genetic-ancestry haplotype that encompasses *WNT5B*. As *GPC4* is found on the X chromosome, our current tool is unable to compute local ancestry on sex chromosomes and we cannot comment further on this relationship.

*PHGDH* has been shown to support metabolism, is required for germline center formation during embryogenesis, and plays an inhibitory role associated with midline defects [69,70]. Affected child 4 carries a predicted damaging homozygous variant in the *PHGDH* gene (rs143340742). Furthermore, the locus of this gene is within a copy-neutral LOH and may suggest that the pathogenic allele copy was made from one parent. The *PHGDH* gene locus switched from heterozygous ancestral structure in parents to homozygous East Asian genetic ancestry in the affected child. The alternate (c.932C > T) T allele frequency is reported as 2/276, 962 in the gnomAD total population, with both coming from 2/24,024 (gnomAD_AFR) from the African population and none reported in the East Asian population, 0/18,868 (gnomAD_EAS), further stressing the importance of not relying on geographical clustering but local ancestry structure.

Copy-number analyses detected copy losses in *ARFRP1*, *CEP290*, *RGS18*, and *TMTC3* genes in affected child 20. The true nature of these copy-number changes is difficult to assess without functional validation. Many of these genes are essential in ciliogenesis, such as *CEP290*, which is critical for early ciliary formation and transition zone assembly [71], *TMTC3*, which is implicated in centrosome formation that is the foundation of cilium development [72], *GIPC3*, which modulates Hedgehog signaling [73], *RGS18*, which regulates ciliogenesis through the Wnt5b pathway [74], and ARF-related proteins, which have been implicated in cilium function [75]. Although these structural events still maintain one copy of the gene and the true biological assessment of pathogenicity is hard to assess, the copy loss may implicate an impact on protein equilibrium.

To date, this is the first report of genetic work in Madagascar that provides the unique opportunity to identify disease-causing variants from protein-expressing regions of the genome in nCL/P case–parent trios. We have identified multiple genes that may contribute to the risk for nCL/P and are influenced by over transmission of East Asian ancestry-enriched alleles. Collectively, these findings add to our understanding of the Malagasy people and population-specific genetic differences that can contribute to the risk of OFCs.

A better understanding of ancestral impact may allow for improvements in management and may explain hereditary complexities in admixed populations. We are aware that further studies in a larger cohort will be critical to identifying mechanistic implications, the complete phenotypic spectrum, and the penetrance of these variants to improve genetic counseling in admixed families with specific mutation motifs. Additionally, we understand that the use of whole-exome sequencing may not be the most robust method to call copy-number variants, as whole-genome sequencing may be more appropriate. However, to address the whole-exome weakness, we boosted our panel to provide a better copy-number resolution. Lastly, we are aware that further work needs to be conducted to functionally validate the results by transcriptome/proteome or other in vivo models and that this is an observational analysis. We hope that, as scientific interests diversify, there will be more data generated from underrepresented and unique populations to better understand added complexities of multi-ethnic genomes. Studies such as this will help the scientific community to expand our comprehension of allelic transmissions and overall genome impact that, then, will help to better understand the genome complexities of clefting. Specifically, we hope that similar local genetic ancestry analyses will empower us to recognize the hereditary patterns in admixed genomes that improve our insights into the biology of race-specific rates of incidents. This work can help contribute to the necessary foundation for future integrated approaches across other studies to help better understand the molecular pathology of nCL/P.

## 4. Materials and Methods

### 4.1. Ethical Approval

Ethical approval for this study was obtained from the University of Southern California Institutional Review Board under IRB #HS-16-00138. Site-specific authorizations and approvals were additionally collected. Ethical approval was also obtained from the Madagascar Ministry of Health under 123-MSANP/CE. Informed consent was obtained from all participating individuals prior to their inclusion in the study.

### 4.2. Participants

Data for this study were collected from 2016 to 2018 as part of a coordinated series of population-sampled case–control studies focusing on genetics, lifestyle, and environmental exposures with nCL/P in children 6 months to 4 years of age. This study was conducted with Operation Smile (OS), an internationally recognized not-for-profit that has been providing free cleft surgery and related care to patients for over 40 years. The methods of this study have been published in-depth previously [31,76]. Data for the current analysis represent 26 nCL/P case–parent trios from the collections that took place in Madagascar at the Centre Hospitalier de Référence Régional (CHRR), the regional referral hospital in Antsirabe. Participation rates in the study varied by collection from 68 to 96% for eligible trios.

### 4.3. Case Definition

This study includes only non-syndromic cases of cleft lip with or without palate (nCL/P) (ICD10 35–37) [77]. The cleft phenotype is classified as either cleft lip and palate (CLP), isolated cleft lip (iCL), or isolated cleft palate (iCP). Cases were screened to confirm the diagnosis, and absence of any genetic syndrome or birth defect by medical practitioners at the OS program site. This included pediatricians, nurses, anesthesiologists, and surgeons who were all formally licensed, trained, and OS-certified to work with patients with OFCs.

Patients were eligible for the study if they were accompanied by their biological mother (18 years or older), 6 months to 4 years of age, and presented for cleft treatment at the time of the OS program. Patients were excluded if the child was not the most recent pregnancy, had multiple births, had a genetic syndrome, or had another co-morbid condition. For the purposes of this study, patients were only eligible if both parents were present, both parents and the child provided a saliva sample, and neither parent had any craniofacial malformation as well as any family history of CL/P. Additionally, families were only selected to be included in this study if both parents self-identified as members of the Merina ethnic tribe.

### 4.4. Family Data Collection

Data were collected by in-person interviews with the mothers and fathers of cases for all participants in the study. Local volunteers with medical training (i.e., nursing/medical students) were identified by OS and underwent training to conduct the interviews and collect the saliva samples. Informed consent was completed before each interview, and parents were assured that participation was not required for their child to receive care. The interview included detailed information regarding family history for CL/P, environmental exposures, and other medical conditions. Whole-exome sequencing was conducted using DNA from saliva collected from the case children and both parents. All DNA was collected in the form of saliva using Mawi (Mawi DNA Technologies, ISWAB-DNA-250) and DNA Genotek (DNA Genotek, Inc., (Kanata, ON, Canada) OGR-175, OGR-500, OGR-525, and OGR-575) collection kits. The samples were collected using protective measures to eliminate contamination issues.

### 4.5. Whole-Exome Sequencing

The quality and quantity of isolated DNA was measured using the Genomic DNA Screen Tape Assay (Agilent Technologies, Santa Clara, CA, USA). A 200-nanogram measure of DNA was sheared in 50 µL of nuclease-free water with a Covaris E220 using a 96 microTUBE Plate (Covaris, Woburn, MA, USA), followed by SureSelect XT Low Input (Agilent) with unique dual adapters. Adapter-ligated libraries were enriched using a custom Agilent’s SureSelect v6 + UTR + OneSeq LowRes (Agilent, Santa Clara, CA, USA) probe set. These additional custom probes (105 Mb exome) provided (1) evenly spaced probes for more-informative copy-number analysis and (2) probes that tile across non-coding regions for improved ancestry calling. Each library was normalized, pooled, and sequenced on Illumina’s NovaSeq 6000 using the S4 300 cycle flow cell (Illumina, San Diego, CA, USA) v1.0 chemistry. All sequencing reads were converted to industry-standard FASTQ files using BCL2FASTQ v1.8.4.

### 4.6. Primary Data Processing

Data were aligned to GRCh37d5 by BWA (v0.7.8-r455), followed by the use of GATK’s Base Recalibrator (v3.5.0) to detect quality score errors and Picard Tools (v1.128) to merge aligned BAMs and mark duplicate reads. Picard MultiMetrics and Samtools Stats (v1.2) were used to collect multiple classes of metrics. Variants were obtained by GATK’s Haplotype Caller, Samtools MPileUp paired with BCFtools (v1.2), and Freebayes (v1.1.0-6-gf069ec6). SnpEff (v3.6h) was used to annotate and predict gene variant effects.

### 4.7. Nucleotide-Variant Analysis

Variant classification and inheritance were computed using Alissa (v39) Agilent software (https://www.agilent.com/en/product/next-generation-sequencing/clinical-informatics-platform/alissa-interpret-930086, accessed on 14 September 2022). A detailed illustration of the pipeline can be viewed in Supplementary Figure S1. The following annotation source codes were used: the 1000 Genomes Phase 3 release v5, ClinGen CNV Atlas, ClinGen Dosage Sensitivity Map, CIViC, COSMIC release v92, NCBI ClinVar, Database of Genomic Variants, DECIPHER population CNVs v9.23, DECIPHER syndromes, Variants in the ESP6500SI-V2 dataset of the exome sequencing project (ESP), annotated with SeattleSeqAnnotation137, ExAC release 1.0, JAX-CKB™—version 20200925, dbNSFP v3.0b2, dbSNP build 151, and gnomAD release 2.0.2. To ensure the accuracy of a variant, we deployed multiple in silico validation techniques such as: (1) variants had to contain reading depth > 30 (proband), (2) the variant had to have > 95% base Q30, (3) regions difficult to map were excluded, (4) to provide mapping/alignment confidence we performed alignment using BWA (v0.7.8-r455; main pipeline) and Bowtie2 (v2.3.4.3) and Isaac (v2.0) for manual validation on candidate variants, and (5) each final candidate variant was manually validated by review by Integrative Genome Viewer (IGV) [78]. In addition, we implemented the Germline Managed Variant List filter to highlight specific phenotypes such as HP:0410030 (cleft lip), HP:0000175 (cleft palate), HP:0002006 (facial cleft), HP:0000202 (oral cleft), HP:0100333 (unilateral cleft lip), and HP:0100334 (unilateral cleft palate). Case analyses were performed using the validated classification tree for OFCs. A total of 26 patients were run individually with their parents in a trio setting. Each variant was annotated against a series of in silico publicly available tools as part of the pipeline, such as: (1) the relationship of variants with clinical impact (ClinVar) [79]; (2) statistical models to assess pathogenicity in genetic variants LRT [80] and phyloP [81]; (3) to assess variant prediction on protein structure (Mutation Assessor) [82], (Mutation Taster) [83], (PROVEAN) [84], (SIFT) [85], Poly-Phen-2 (PPHP2HumanVar) [86], and (UniProt) [87]; and (4) methods to predict evolutionary sequencing constraints (SiPhy) [88]. Network enrichment analysis was performed in KEGG [89,90] pathway maps and Ingenuity Pathway Analysis (QIAGEN IPA) [53] using the mutation-level genetic variant data.

Functionally impactful variants predicted to be disease-causing with known Mendelian inheritance mode were prioritized. Considerations were made by filtering variants for reading quality, aligned read depth of coverage predicted functional effect of the variant, inheritance mode across trios, and prevalence of the variants in known population databases. Variants that were reported in the population database gnomAD (alleleFrequencyAll) with less than 0.01% were considered rare. Variants with read depth above 50, a genotype with at least a quality score of 99, a population frequency of less than 0.001%, and with damaging prediction by at least two of the variant prediction tools (mentioned above) were considered for further review. Pathogenicity was determined by Clinvar, LRT, and phyloP score databases. Lastly, a primary literature review was utilized to examine the variant impact on biological processes.

### 4.8. Copy-Number-Variant Analysis

Copy-number-variant (CNV) analysis was performed using Nexus Copy Number10 (https://www.biodiscovery.com/products/nexus-copy-number, accessed on 5 February 2023) (BioDiscovery, El Segundo, CA, USA). To increase the accuracy of CNV calling, we implemented a larger WES probe set as described in Whole-Exome Sequencing that provided an even distribution of probes across the whole genome. Since a Madagascar reference is not widely available to correct for ancestry-specific variations, we built an internal reference from BAMs generated by unaffected control parents (*n* = 52 samples), utilizing the BAM Multiscale Reference Builder (BioDiscovery, El Segundo, CA, USA). We used the “entire genome (no masking)” option to generate copy-number estimations and left the “Minimum Bin Width”, “Average Read Length”, “Target Reads Per Bin”, and “Maximum Neighbor Bin Gap” options as default per software recommendations (Nexus10; BioDiscovery, El Segundo, CA, USA). The 1000 Genome project structural variant file was used as an additional population anchor [91,92]. The software package provides a relative quality control score that assesses probe-to-probe variance. It computed average variations between the magnitudes of successive probes. This study’s average QC score was 0.019 (min = 0.012; max = 0.053; software recommendation QC < 0.2). To ensure the accuracy of copy-number-variant calling, we used the following B-Allele-Frequency (BAF) parameters: (1) reject reads with <20 depth, (2) reject reads with <30 mapping quality (MAPQ), and (3) reject bases with <20 base quality. Gain/Loss cut-off was set at >0.2 or higher with the minimum number of probes per segment set at >3 probes. Focal statistical analysis was performed using a modified approach by the Genomic Identification of Significant Targets in Cancer (GISTIC) package as part of Nexus10 (BioDiscovery) toolkit using stringent cutoffs of G-Score >5 (measurement of the magnitude of the copy-number change and frequency of occurrences) and Q-bound <0.01 (FDR correction for multiple testing) with 100% overlap. The familiar relationship was performed by applying family factors and performing a family (trio) filter according to Nexus10 (BioDiscovery) software recommendations.

### 4.9. Sample QC and Ancestry Analyses

To identify any possible sample mismatches, we performed gender testing by computing chromosome X and Y zygosity. For processing contamination detections, we performed a software tool, verifyBamID [93]. Global ancestry was computed using gVCF using VCFtools (v0.1.17) [94], PLINK (v1.90b6.7) [95], PGDSpider (v2.1.1.5) [96], and STRUCTURE (v2.3.4) [97,98]. STRUCTURE was performed using 3 repeats, k = 4, BURNING = 50,000, and NUMREP = 100,000. The anchor population used was derived from 1000 Genomes project Phase 3 genotype data. Local ancestry was computed using LAMP (v2.5) [99] using populations = 3, recombrate = 10^−8^, generations = 20, ldcutoff = 0.1, offset = 0.2, and was performed on autosomal chromosomes. Visualization was performed using R (v3.6.0): ggplot2 (v3.3.3), ggfortify (v0.4.11), plyr (v1.8.6), dplyr (v1.0.5), and ggrepel (v0.9.1). For visualization of variants, we used Integrative Genome Viewer (IGV v2.9.4) [78] with BAM file inputs.

### 4.10. Statistical Ancestry Analyses

Assessment of genetic variants associated with nCL/P was performed as a child (case) vs. unaffected parents in a family-based case–control association approach. The Chi-square test was used to evaluate the local ancestry equivalence of two proportions (p1 (Asian) = p2 (African)). Significance was determined as *p* < 0.05. To ensure that analyses were performed at the highest quality, we detected SNPs with a high frequency of missing values in either case or control and SNPs that depart from Hardy–Weinberg equilibrium. Focal copy-number-variant (CNV) analysis was performed using GISTIC in Nexus 10. The significant factors were set to be stringent to exclude any population biases, with the FDR-adjusted q-bound set as significant at <0.001 and G-score set at 5.

## 5. Author Summary

Our study is the first of its kind in Malagasy cases with nCL/P, demonstrating a need to examine ancestorial admixture to categorize this congenital malformation better. Adding global genetic ancestry may help provide meaningful molecular answers to population differences reported among nCL/P as well as improve care and management for patients with OFCs who have the highest likelihood of not receiving timely surgical treatment.

## Figures and Tables

**Table 1:** General overview of the project.

Trio Complete	Count	%
Yes	26	100%
Proband Gender		
Male	13	50%
Female	13	50%
Cleft type		
Cleft lip and palate (unilateral)	16	62%
Cleft lip and palate (bilateral)	2	8%
Isolated cleft lip (unilateral)	8	31%
Isolated cleft lip (bilateral)	0	0%
	Avg	Range
Estimated coverage	172.1	147–199
Target bases 10×	95%	89–98%

**Table 2:** Variant description found in 26 Malagasy children with nCL/P.

FamilyID	Child015	Child015	Child005	Child006	Child009	Child023	Child005	Child019	Child004	Child019
**Gene**	WNT5B	GPC4	MSX1	MSX1	MSX1	MSX1	SEPTIN9	WDR11	PHGDH	SKI
**Chromosome**	12	X	4	4	4	4	17	10	1	1
**Start**	1742006	132440095	4861877	4861877	4861877	4861877	75484846	122664299	120279876	2160660
**Stop**	1742006	132440095	4861877	4861877	4861877	4861877	75484846	122664299	120279876	2160660
**Read** **depth**	123	169	89	66	82	81	131	51	131	82
**Reference**	G	G	A	A	A	A	G	A	C	G
**Genotype** **(Proband)**	G|T	C|G	T|A	T|A	T|A	T|A	A|G	G|A	T|T	G|A
**Genotype** **(Mother)**	G|G	G|C	A|T	A|T	A|T	A|T	G|A	A|G	C|T	G|G
**Genotype** **(Father)**	G|T	G|G	A|A	A|A	A|A	A|A	G|G	A|T	C|T	G|A
**Inheritance** **mode**	Heterozygous	Heterozygous	Heterozygous	Heterozygous	Heterozygous	Heterozygous	Heterozygous	Heterozygous	Homozygous	Heterozygous
**Inherited** **from**	Father	Mother	Mother	Mother	Mother	Mother	Mother	Mother	Both	Father
**cDNA**	c.263G > T	c.965C > G	c.251A > T	c.251A > T	c.251A > T	c.251A > T	c.1108G > A	c.3169A > G	c.932C > T	c.455G > A
**HGVS**	p.Arg88Leu	p.Ala322Gly	p.Glu84Val	p.Glu84Val	p.Glu84Val	p.Glu84Val	p.Glu370Lys	p.Met1057Val	p.Ser311Phe	p.Arg152His
**Exon**	3	5	1	1	1	1	6	25	8	1
**dbSNP**	rs200966877		rs28928890	rs28928890	rs28928890	rs28928890	rs1297513860		rs143340742	
**OMIM(r) refs**	606361						604061			
**clinVar ClinicalSig**			pathogenic	pathogenic	pathogenic	pathogenic				
**clinVar Gene Disease**			Orofacial cleft 5	Orofacial cleft 5	Orofacial cleft 5	Orofacial cleft 5				
**ClinVar ID**			14883	14883	14883	14883				
**LRT prediction**	Deleterious	Deleterious	Neutral	Neutral	Neutral	Neutral	Deleterious	Deleterious	Neutral	Deliterious
**Mutation Assessor** **Prediction**	Medium	Medium	Low	Low	Low	Low	Medium	Medium	Low	Medium
**Mutation Taster Prediction**	Disease causing	Disease causing	Disease causing automatic	Disease causing automatic	Disease causing automatic	Disease causing automatic	Disease causing	Disease causing	Disease causing	Disease causing
**phyloP**	0.917	0.917	1.039	1.039	1.039	1.039	0.913	1.062	0.871	0.917
**PPH2HumVar Prediction**	Benign	Probably damaging	Benign	Benign	Benign	Benign	Probably damaging	Possibly damaging	Possibly damaging	Probably damaging
**PROVEAN** **Prediction**	Damaging	Damaging	Neutral	Neutral	Neutral	Neutral	Damaging	Neutral	Damaging	Damaging
**SIFT score**	0.025	0.058	0.102	0.102	0.102	0.102	0.002	0.013	0.005	0.001
**SiPhyScore**	12.313	17.852	10.831	10.831	10.831	10.831	15.005	16.461	14.386	15.381
**gnomAD_AF** **All**	1/246220 = 0		1/102294 = 0	1/102294 = 0	1/102294 = 0	1/102294 = 0	1/246242 = 0		2/276962 = 0	
**gnomAD_AF** **AMR**	0/33580 = 0		0/19614 = 0	0/19614 = 0	0/19614 = 0	0/19614 = 0	0/33578 = 0		0/34414 = 0	
**gnomAD_AF** **AFR**	1/15304 = 0		1/2042 = 0	1/2042 = 0	1/2042 = 0	1/2042 = 0	0/15302 = 0		2/24024 = 0	
**gnomAD_AF** **ASJ**	0/9848 = 0		0/6996 = 0	0/6996 = 0	0/6996 = 0	0/6996 = 0	0/9850 = 0		0/10142 = 0	
**gnomAD_AF** **EAS**	0/17248 = 0		0/6594 = 0	0/6594 = 0	0/6594 = 0	0/6594 = 0	0/17248 = 0		0/18868 = 0	
**gnomAD_AF** **FIN**	0/22284 = 0		0/6182 = 0	0/6182 = 0	0/6182 = 0	0/6182 = 0	0/22292 = 0		0/25718 = 0	
**gnomAD_AF** **NFE**	0/111690 = 0		0/38366 = 0	0/38366 = 0	0/38366 = 0	0/38366 = 0	1/111704 = 0		0/126562 = 0	
**gnomAD_AF** **OTH**	0/5484 = 0		0/2888 = 0	0/2888 = 0	0/2888 = 0	0/2888 = 0	0/5486 = 0		0/6456 = 0	
**gnomAD_AF** **SAS**	0/30782 = 0		0/19612 = 0	0/19612 = 0	0/19612 = 0	0/19612 = 0	0/30782 = 0		0/30778 = 0	

## Data Availability

The data presented in this study are available on request from the corresponding author. The data are not publicly available due to privacy restrictions.

## Supplementary Materials

The following supporting information can be downloaded at: https://www.mdpi.com/article/10.3390/genes14030665/s1, Supplementary Figure S1. Alisa (Agilent Inc.) pipeline schematic outline. A visual representation of the variant annotation pipeline used in this study. Supplementary Figure S2. Local ancestry was calculated across the remainder of the families. Genome-wide local ancestry was calculated by the LAMP tool for the remainder of the families in this study. The child has nCL/P birth defects; the mother and father have no history of craniofacial abnormalities. The x-axis shows the chromosome distribution; the y-axis shows the relative size of each haplotype. Yellow indicates East Asian ancestry, blue indicates African ancestry, and red indicates South Asian ancestry contributions. Each set of data is generated per individual for each family. Boxes with gene names indicate the relative position of the gene of interest. Supplementary Figure S3. Loss of heterozygosity in *PHGDH* in Family 4 with nCL/P. Gene-level copy-number analysis is performed by Nexus 10 (Biodiscovery). The position of focus is the *PHGDH* locus (black error). The proband has a unilateral cleft lip and palate phenotype, and the parents (control) have no family history of CL/P. The left panel indicates copy-number state in the child, the middle in the mother, and the right in the father. The top box shows copy-number log ratios, with value = 0 indicating copy neutrality. The bottom panels across indicate b-allele frequencies. The child (yellow) shows a copy of neutral LOH. Supplementary Figure S4. Focal copy-number analysis by GISTIC to evaluate proband and control parents. Here, we show a GISTIC plot across the proband (case) and parents (control). The (red) indicates copy-number loss, and (blue) indicates copy-number gain for each stratified group. Statistical value is presented on the x-axis as q-bound, and chromosome events were shown across the y-axis. Each major peak highlighted by a gray line shows significance (q-bound < 0.001; G-score > 5).
