# Supplementary material for: Genome Analysis Using Whole-Exome Sequencing of Non-Syndromic Cleft Lip and/or Palate from Malagasy Trios Identifies Variants Associated with Cilium-Related Pathways and Asian Genetic Ancestry
Source: Genes (Basel). 2023 Mar 7;14(3):665. doi: 10.3390/genes14030665 (PMC10048728; doi:10.3390/genes14030665)
Supplement: Supplementary file 1 [file genes-14-00665-s001.zip › genes-2235229-supplementary.pdf]

Supplementary Figure S1

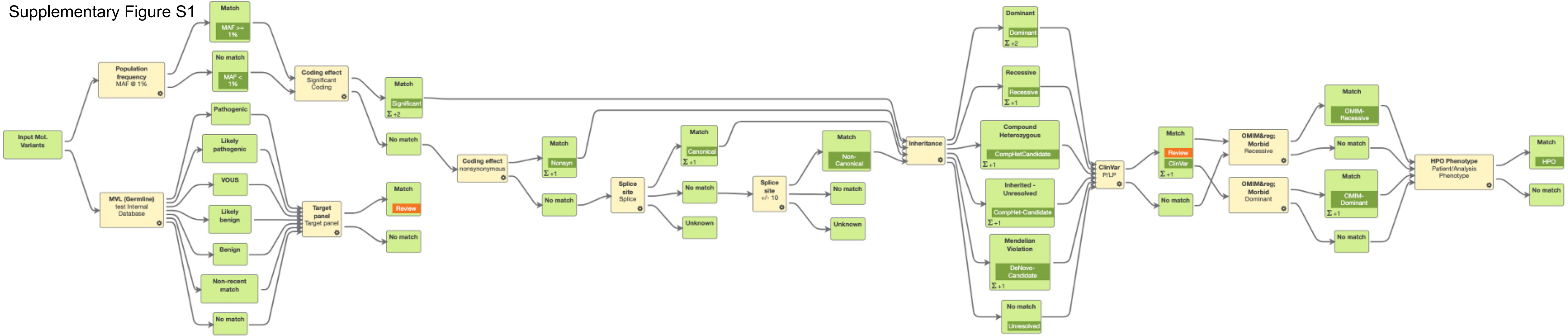

Supplementary Figure S2

Family_001

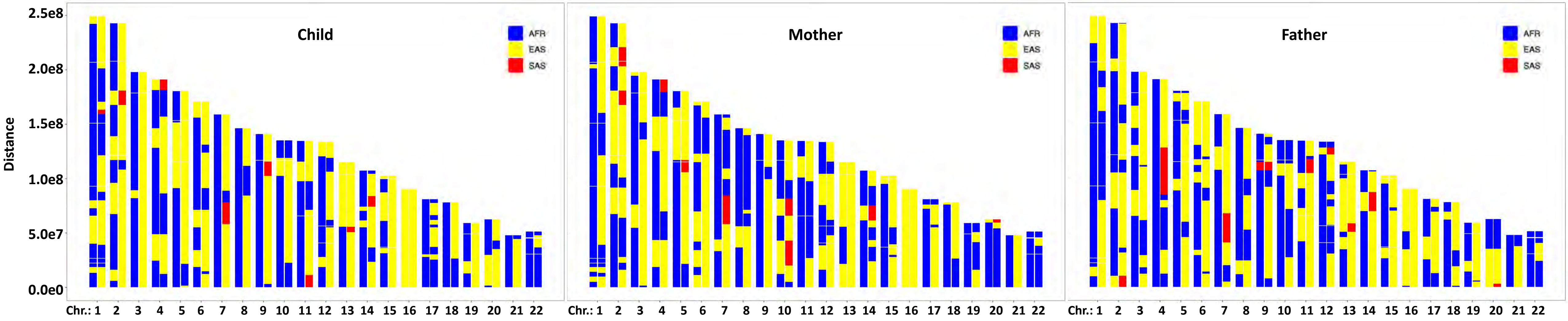

Family_002

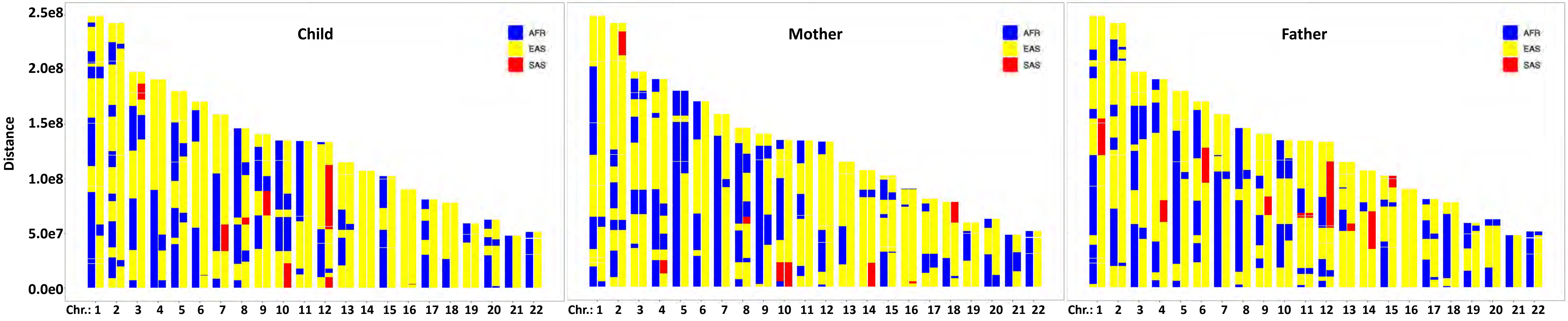

Family_003

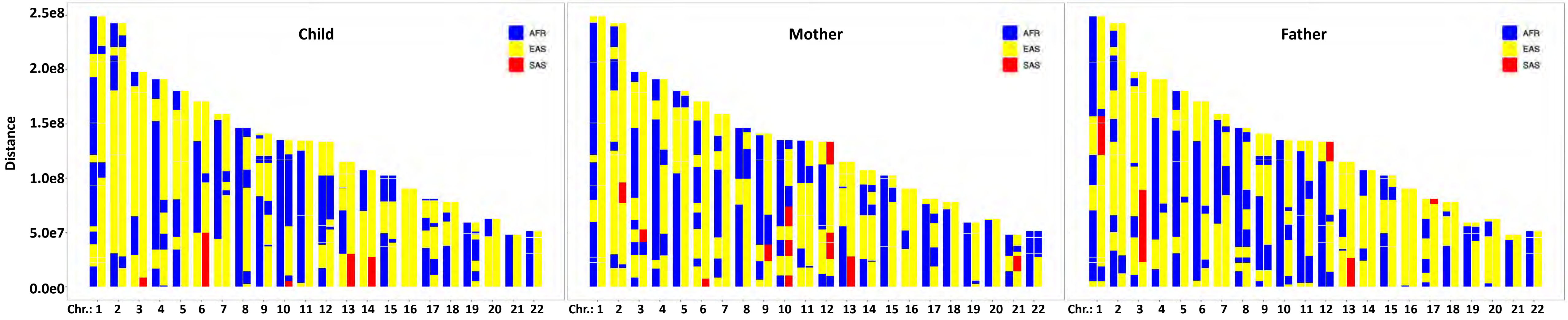

Family_004

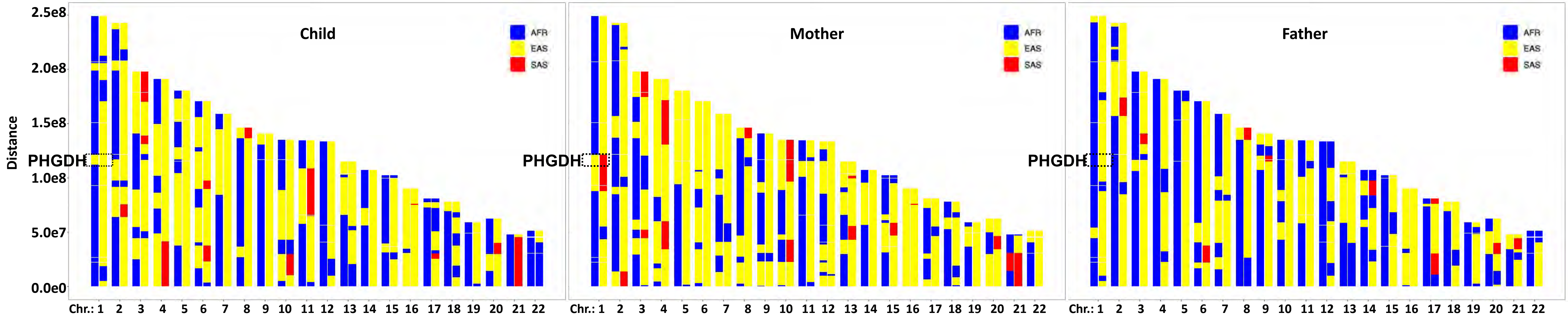

Family_005

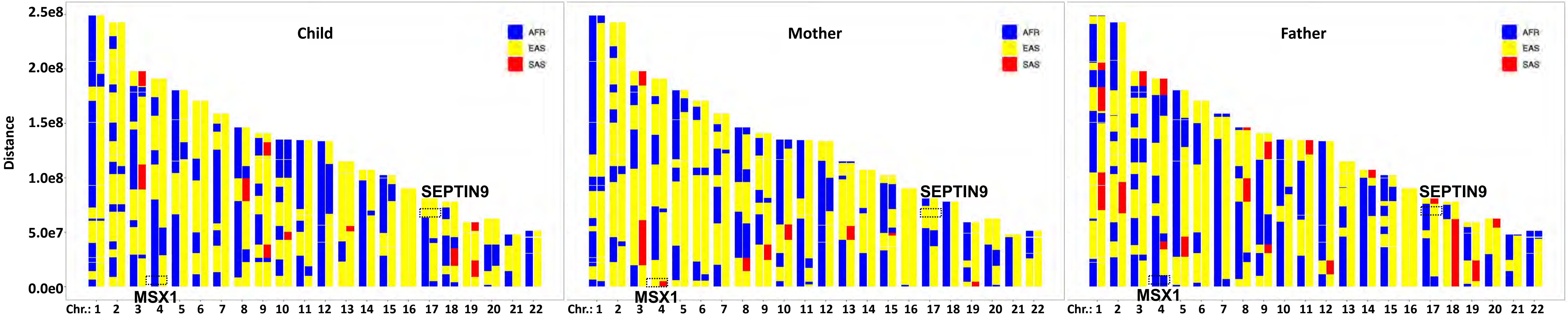

Family_006

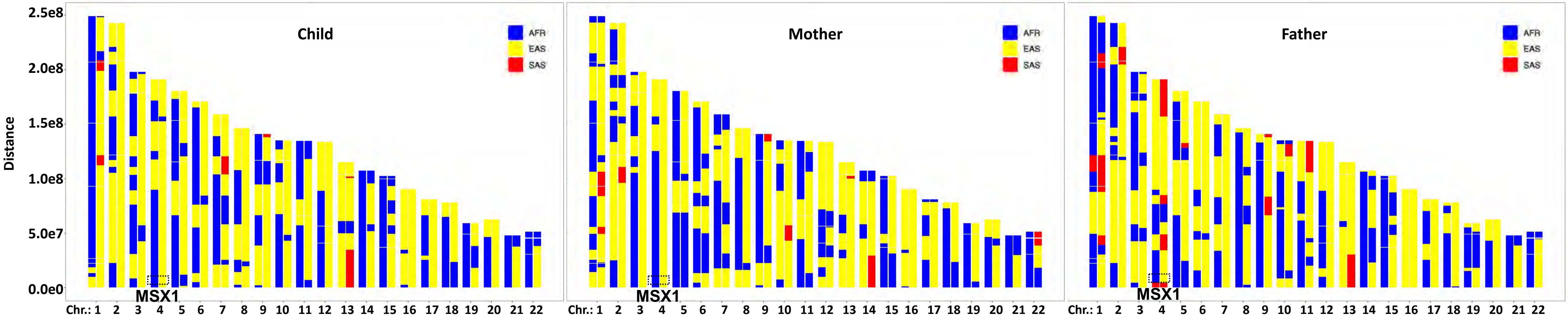

Family_007

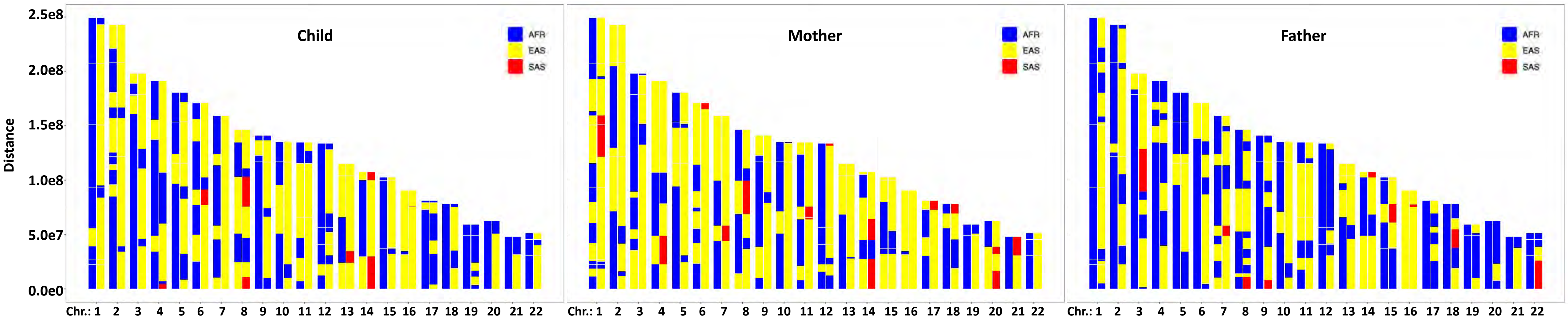

Family_008

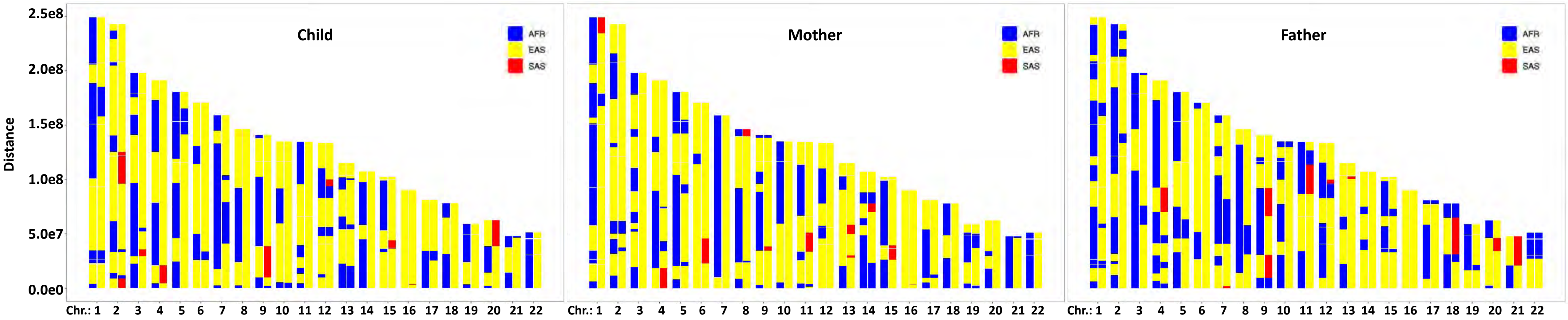

Family_009

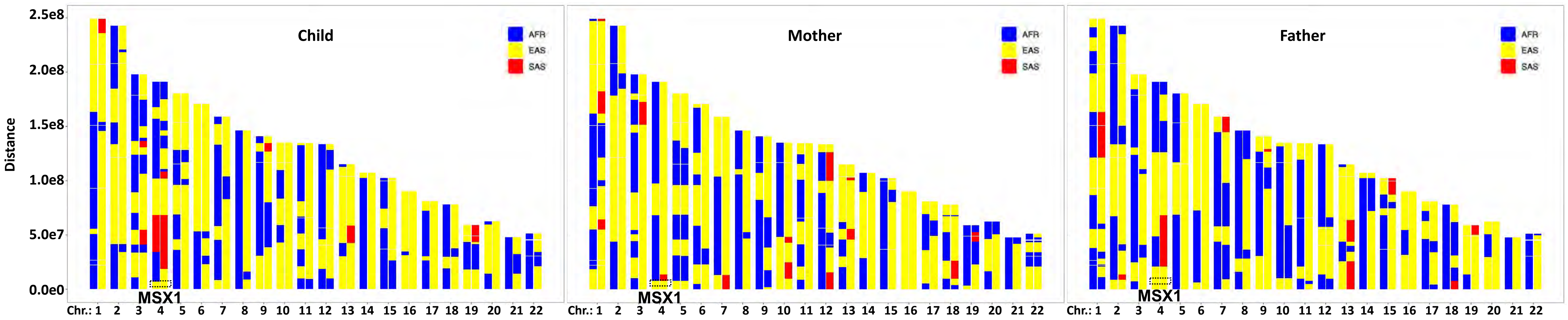

Family_010

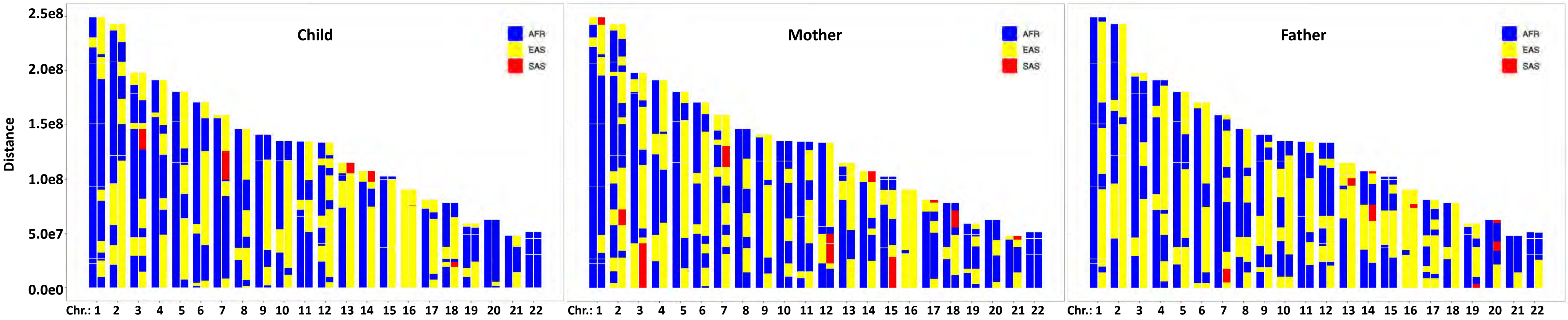

Family_011

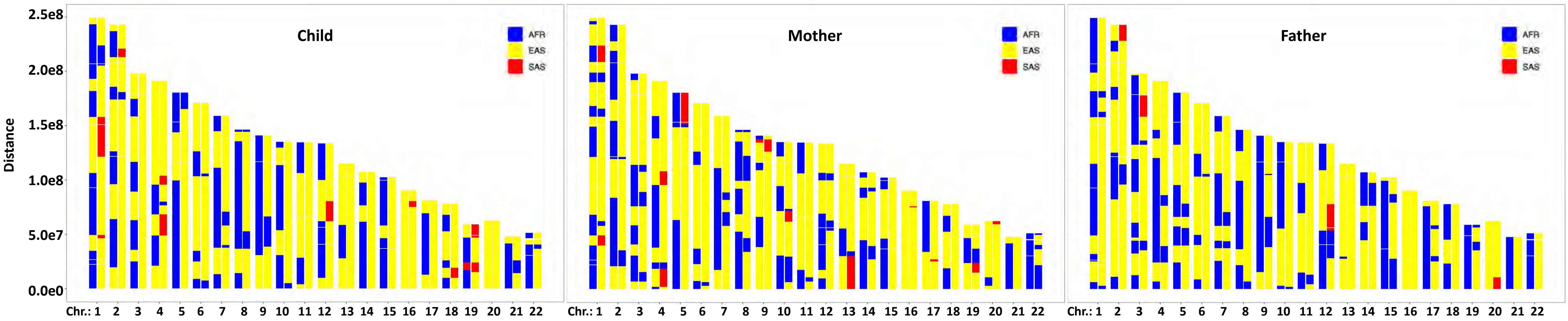

Family_012

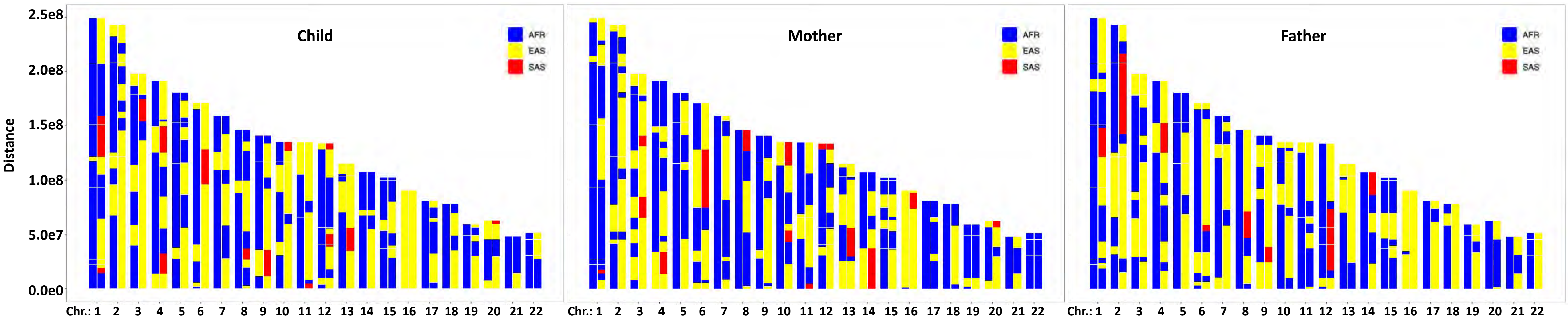

Family_013

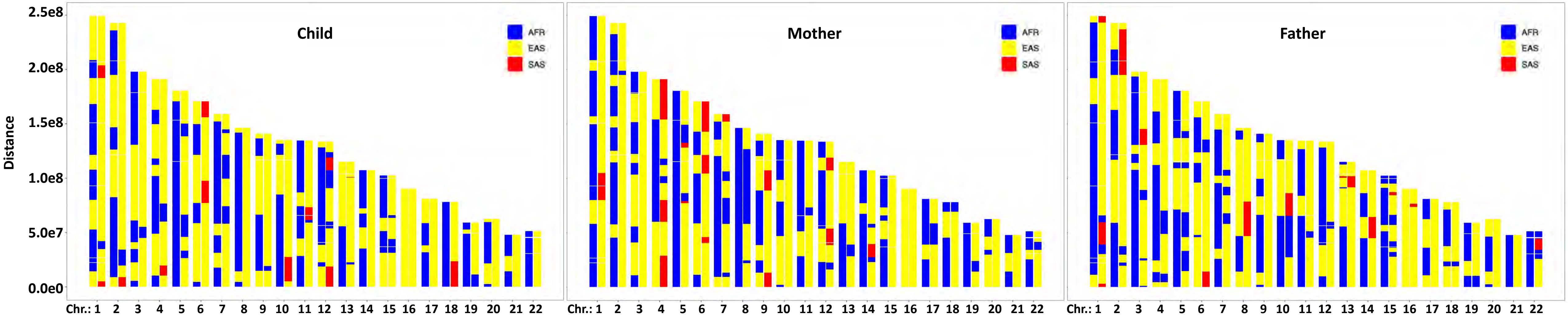

Family_014

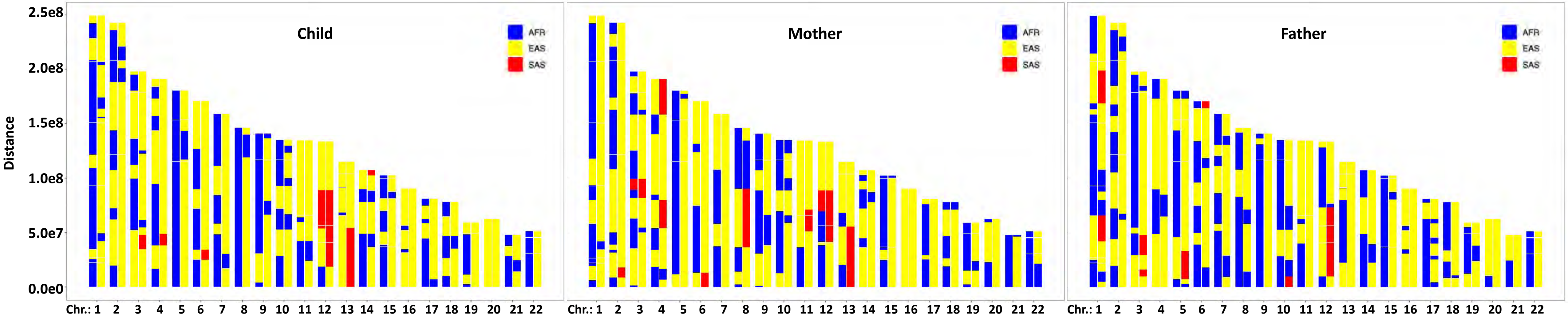

Family_015

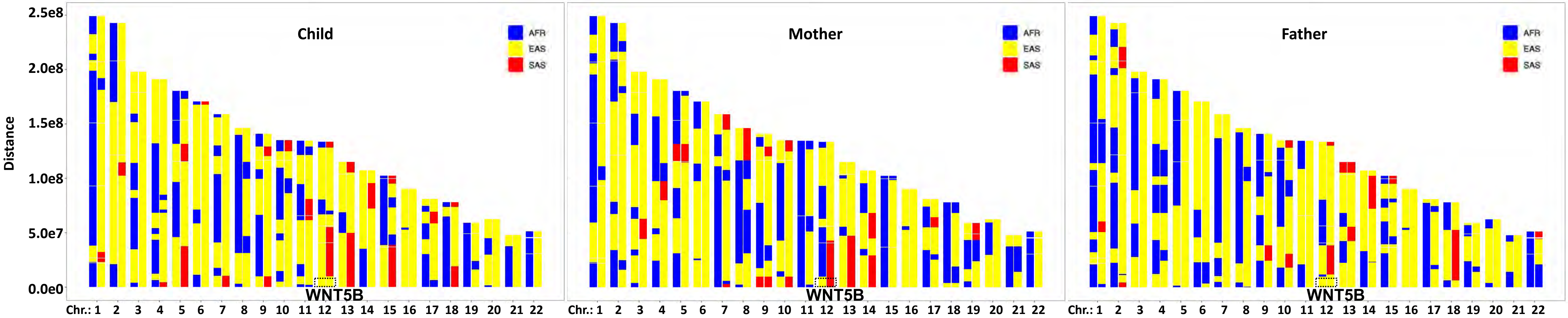

Family_016

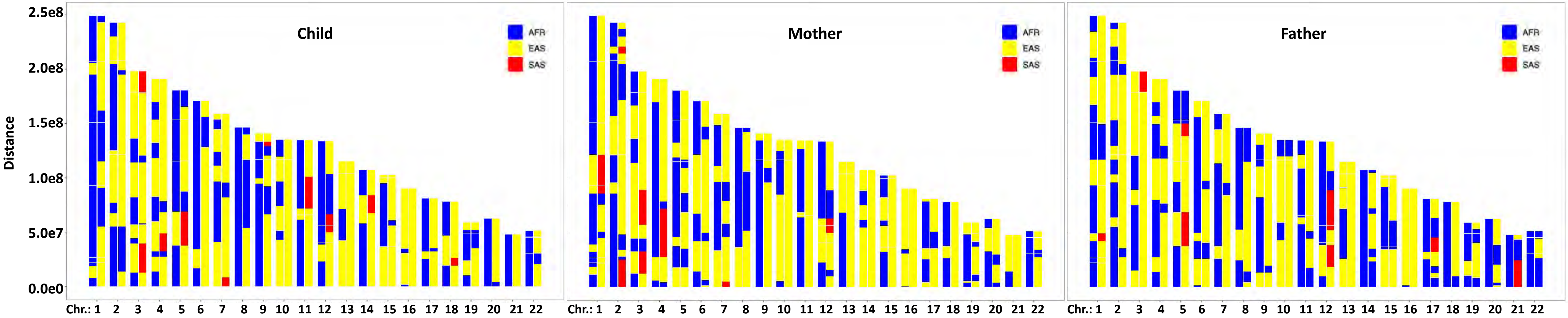

Family_017

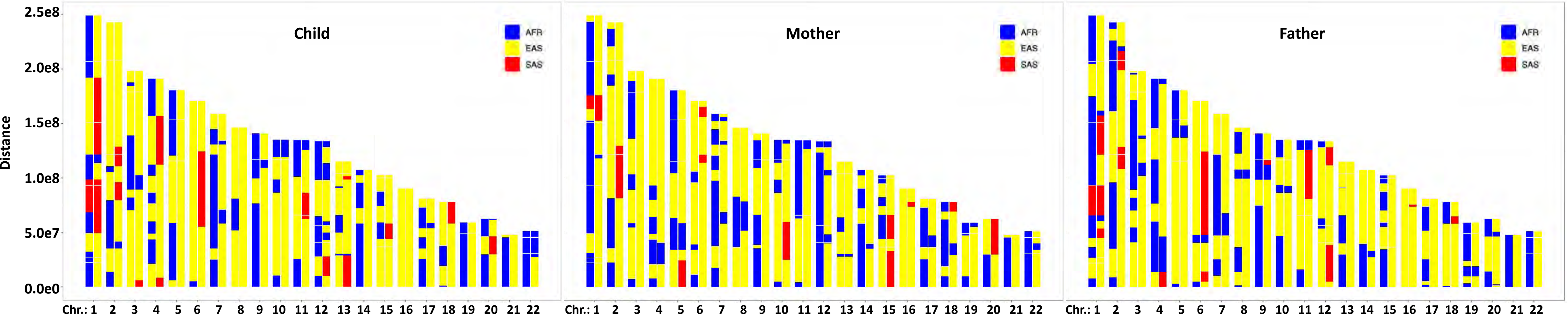

Family_018

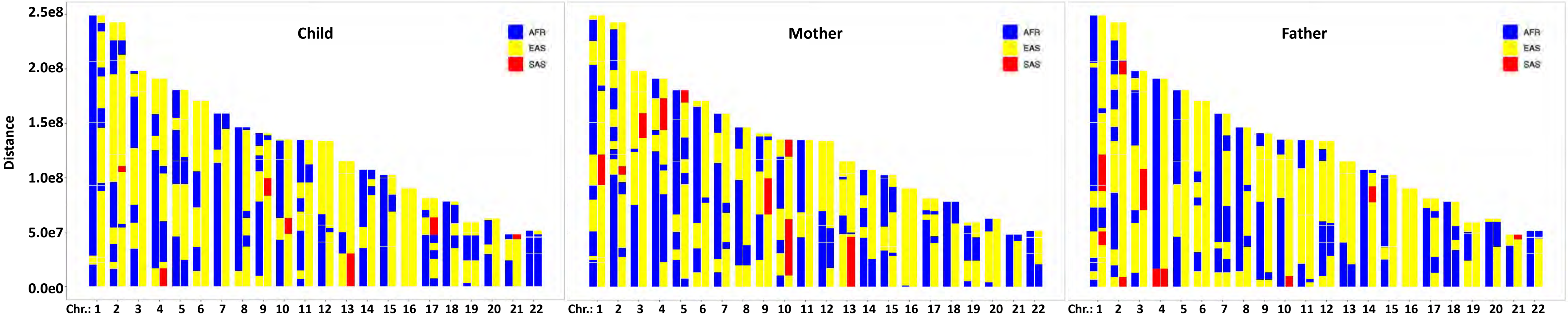

Family_019

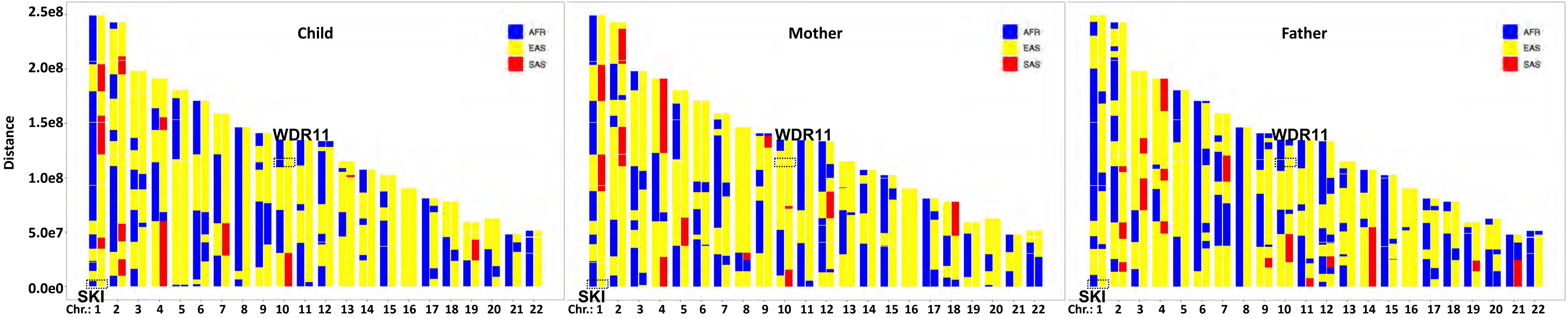

Family_020

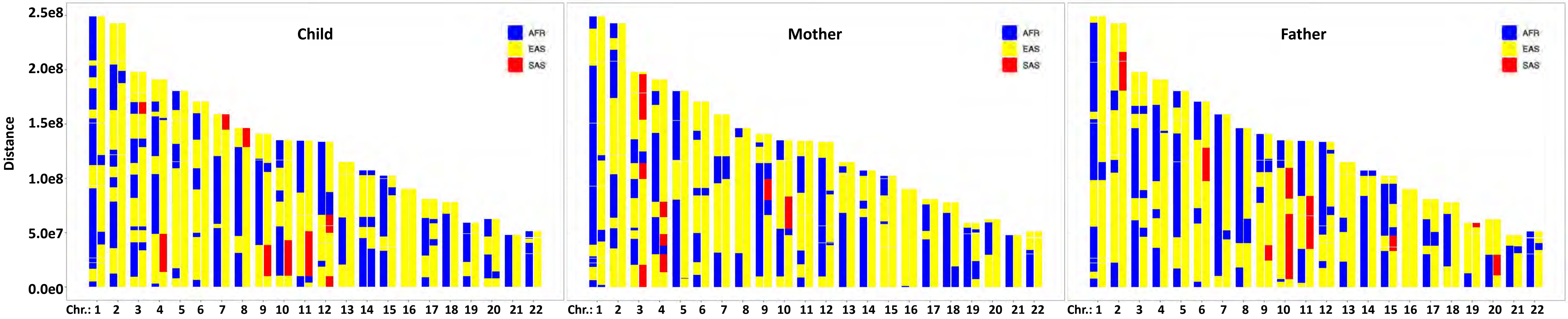

Family_021

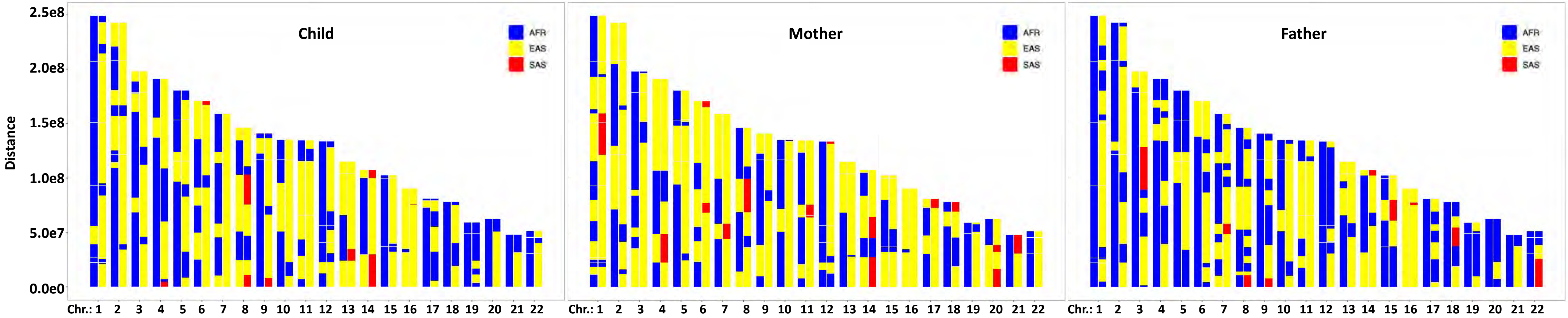

Family_022

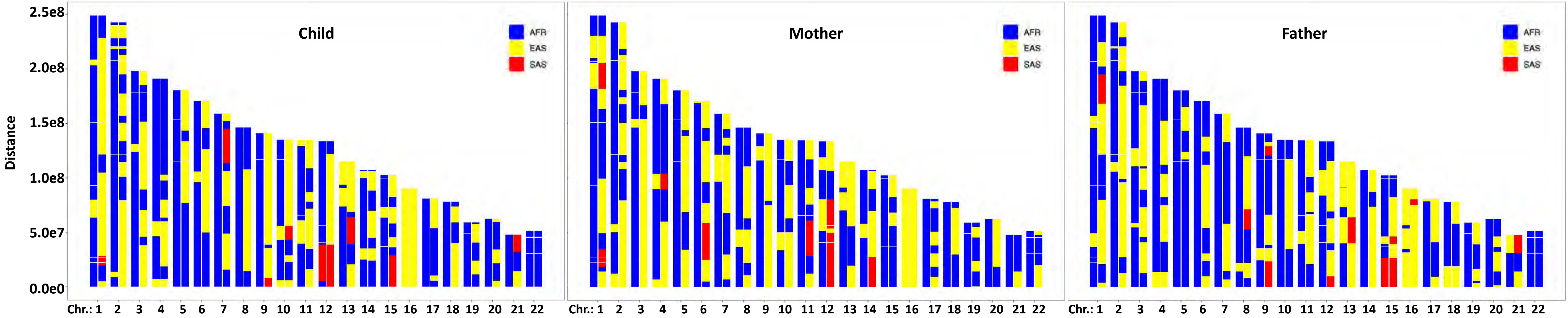

Family_023

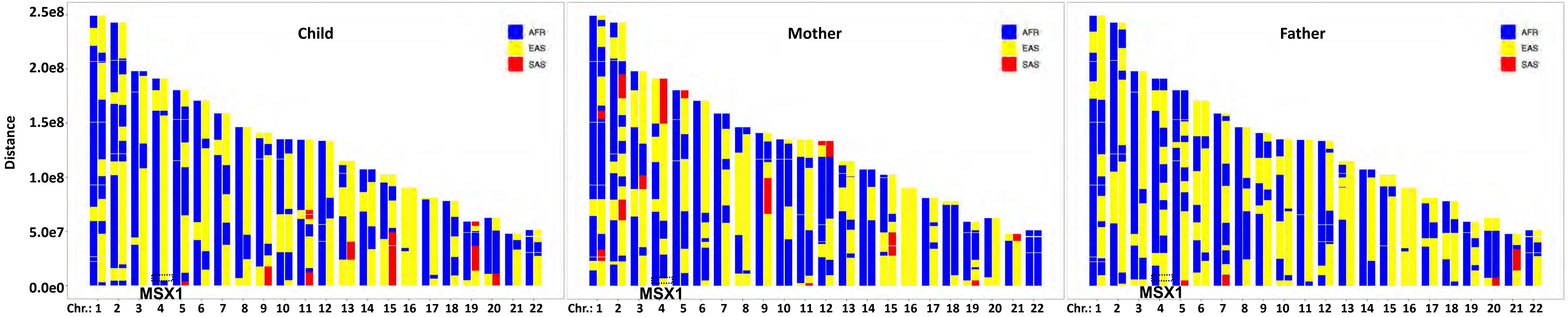

Family_024

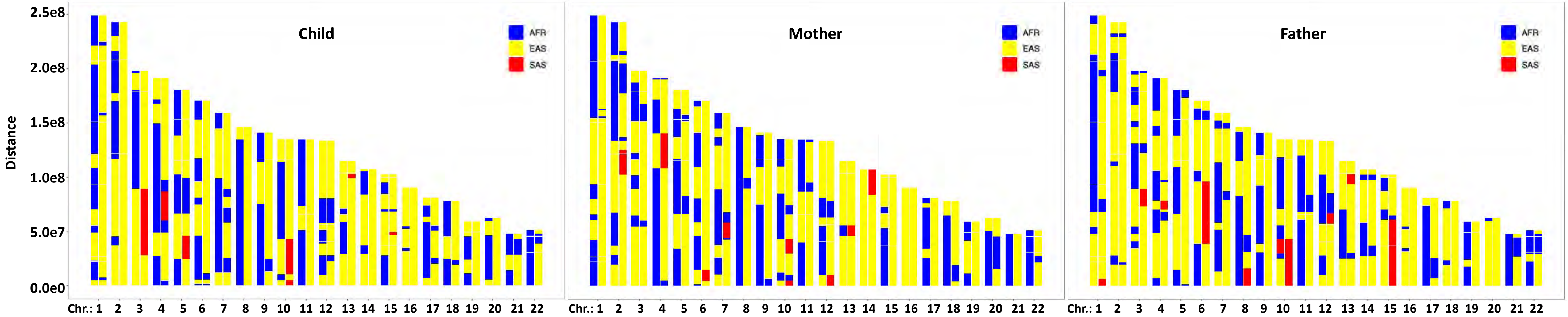

Family_025

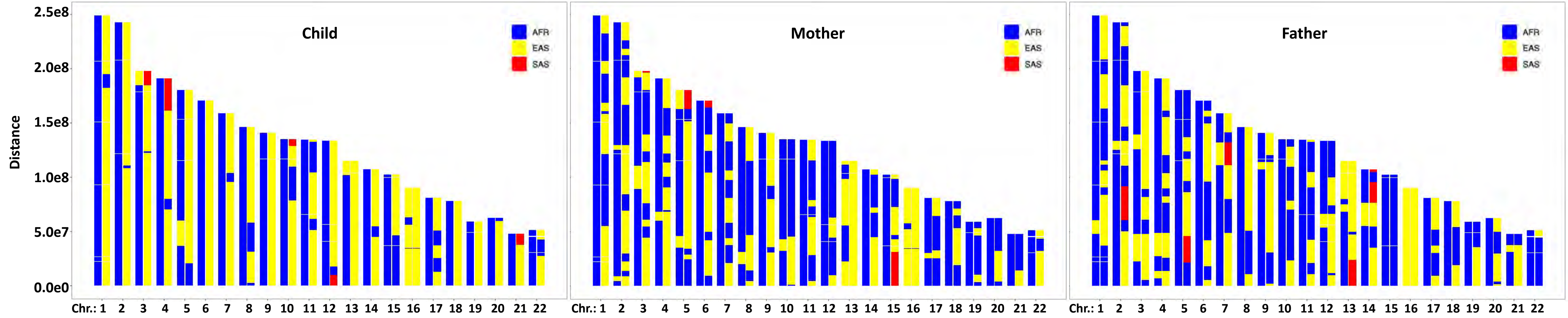

Family_027

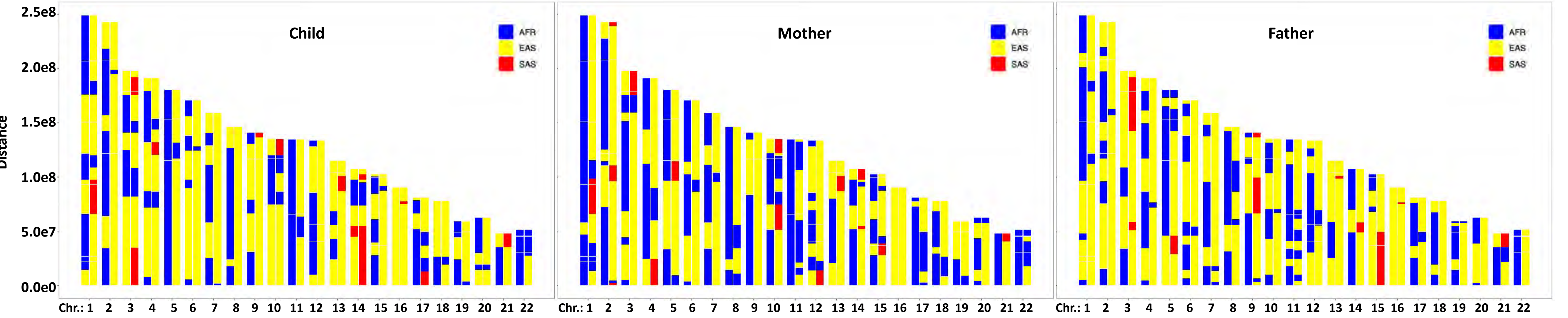

Supplementary Figure S3

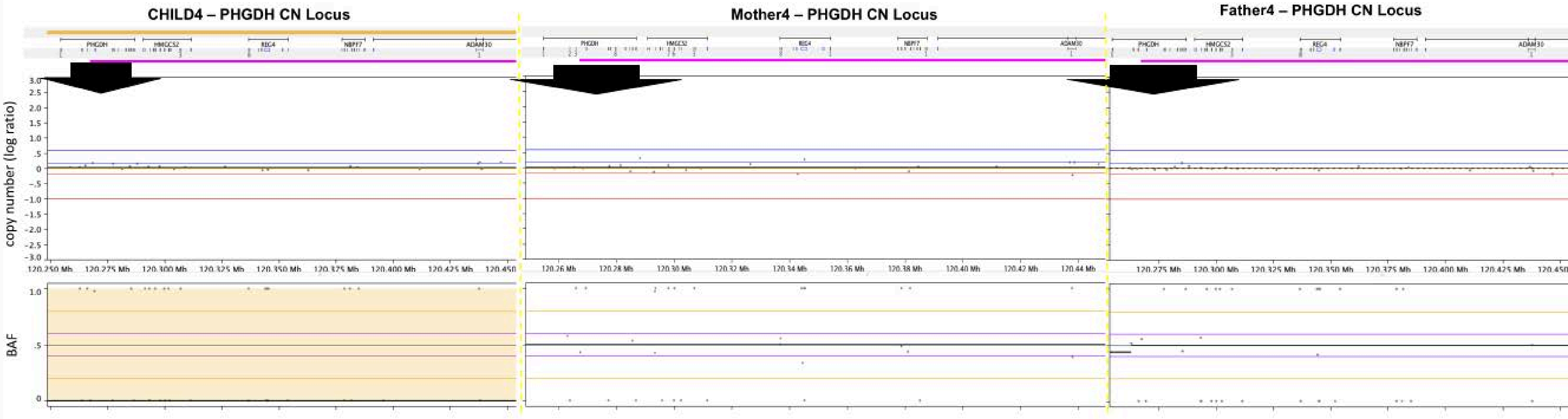

Supplementary Figure S4

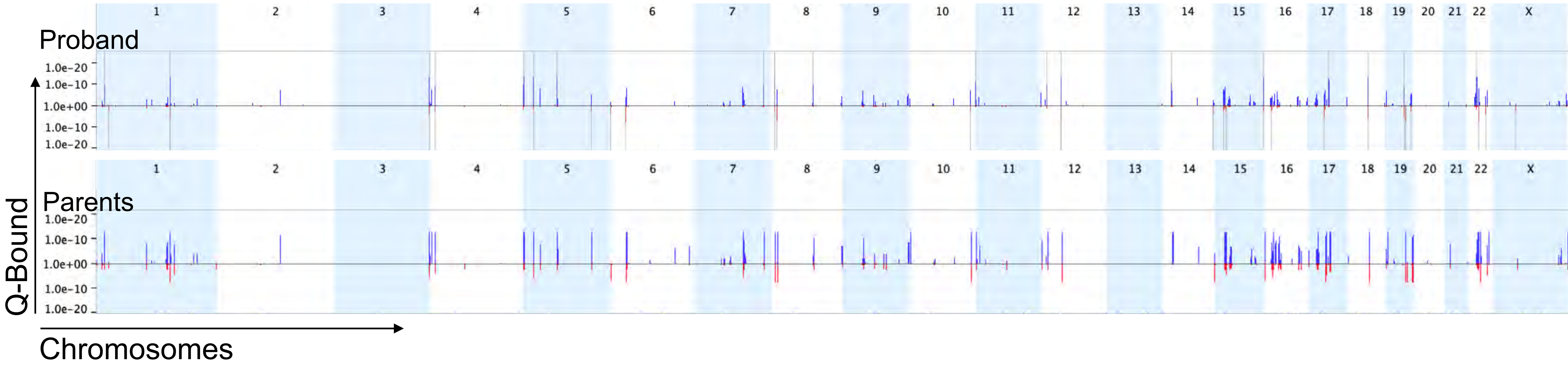

Supplementary Table S1. General Overview of the Project.

Sample_ID	Family_ID	Gender	Clinical Phenotype	Sequencing_ID	M Reads Mapped	% Dups	Yield (Mb)	Estimated Coverage	Target Bases 10X
63450	MOTHER1	Female	Negative	KOPSMCHBM_0001_01_MOTHER01_R00201S8A2M0001P0000_C1_AV6UOS_A00960	98.3	10.90%	15,973	160	91%
61290	FATHER1	Male	Negative	KOPSMCHBM_0002_01_FATHER01_R00201S8A2M0000P0001_C1_AV6UOS_A00961	107.2	12.00%	16,349	163	93%
61780	CHILD1	Female	UCL/P (unilateral cleft lip and palate)	KOPSMCHBM_0003_01_CHILD001_R00201S8A2M0000P0000_C1_AV6UOS_A00962	120.1	12.40%	18,931	189	94%
61600	CHILD2	Female	UCL/P (unilateral cleft lip and palate)	KOPSMCHBM_0004_01_CHILD002_R00202S8A2M0000P0000_C1_AV6UOS_A00963	127.4	14.20%	19,908	199	96%
61495	FATHER2	Male	Negative	KOPSMCHBM_0005_01_FATHER02_R00202S8A2M0000P0001_C1_AV6UOS_A00964	117.1	12.00%	18,019	180	93%
61494	MOTHER2	Female	Negative	KOPSMCHBM_0006_01_MOTHER02_R00202S8A2M0001P0000_C1_AV6UOS_A00965	108.7	12.10%	16,862	169	94%
61772	CHILD3	Female	UCL (unilateral cleft lip)	KOPSMCHBM_0007_01_CHILD003_R00203S8A2M0000P0000_C1_AV6UOS_A00966	105	11.80%	16,877	169	94%
61374	MOTHER3	Female	Negative	KOPSMCHBM_0008_01_MOTHER03_R00203S8A2M0001P0000_C1_AV6UOS_A00967	113.3	11.20%	17,190	172	93%
61373	FATHER3	Male	Negative	KOPSMCHBM_0009_01_FATHER03_R00203S8A2M0000P0001_C1_AV6UOS_A00968	102.9	11.50%	16,887	169	92%
64005	CHILD4	Female	UCL/P (unilateral cleft lip and palate)	KOPSMCHBM_0010_01_CHILD004_R00204S8A2M0000P0000_C1_AV6UOS_A00969	123	12.30%	18,578	186	96%
63536	FATHER4	Male	Negative	KOPSMCHBM_0011_01_FATHER04_R00204S8A2M0000P0001_C1_AV6UOS_A00970	114.8	12.60%	17,389	174	96%
63702	MOTHER4	Female	Negative	KOPSMCHBM_0012_01_MOTHER04_R00204S8A2M0001P0000_C1_AV6UOS_A00971	95.9	11.00%	14,701	147	93%
81700	CHILD5	Male	UCL (unilateral cleft lip)	KOPSMCHBM_0013_01_CHILD005_R00205S8A2M0000P0000_C1_AV6UOS_A00972	111.3	13.50%	17,547	175	96%
68019	FATHER5	Male	Negative	KOPSMCHBM_0014_01_FATHER05_R00205S8A2M0000P0001_C1_AV6UOS_A00973	105.1	12.40%	16,242	162	94%
68046	MOTHER5	Female	Negative	KOPSMCHBM_0015_01_MOTHER05_R00205S8A2M0001P0000_C1_AV6UOS_A00974	109.3	11.70%	16,746	167	94%
82207	CHILD6	Male	UCL/P (unilateral cleft lip and palate)	KOPSMCHBM_0016_01_CHILD006_R00206S8A2M0000P0000_C1_AV6UOS_A00975	112.5	11.50%	17,052	171	94%
63713	MOTHER6	Female	Negative	KOPSMCHBM_0017_01_MOTHER06_R00206S8A2M0001P0000_C1_AV6UOS_A00976	94.6	11.60%	14,851	149	94%
66533	FATHER6	Male	Negative	KOPSMCHBM_0018_01_FATHER06_R00206S8A2M0000P0001_C1_AV6UOS_A00977	107.9	10.70%	16,784	168	91%
61516	CHILD7	Male	UCL/P (unilateral cleft lip and palate)	KOPSMCHBM_0019_01_CHILD007_R00207S8A2M0000P0000_C1_AV6UOS_A00978	101.2	12.00%	15,659	157	95%
61210	MOTHER7	Female	Negative	KOPSMCHBM_0020_01_MOTHER07_R00207S8A2M0001P0000_C1_AV6UOS_A00979	105	12.80%	16,136	161	96%
61213	FATHER7	Male	Negative	KOPSMCHBM_0021_01_FATHER07_R00207S8A2M0000P0001_C1_AV6UOS_A00980	99.2	12.20%	15,079	151	94%
63629	MOTHER8	Female	Negative	KOPSMCHBM_0022_01_MOTHER08_R00208S8A2M0001P0000_C1_AV6UOS_A00981	108.7	11.90%	16,981	170	95%
81695	CHILD8	Male	UCL/P (unilateral cleft lip and palate)	KOPSMCHBM_0023_01_CHILD008_R00208S8A2M0000P0000_C1_AV6UOS_A00982	118.6	12.70%	18,353	184	95%
63715	FATHER8	Male	Negative	KOPSMCHBM_0024_01_FATHER08_R00208S8A2M0000P0001_C1_AV6UOS_A00983	114.9	13.40%	18,716	187	95%
68037	FATHER9	Male	Negative	KOPSMCHBM_0025_01_FATHER09_R00209S8A2M0000P0001_C1_AV6UOS_A00984	115.2	11.50%	18,011	180	92%
81716	CHILD9	Male	UCL/P (unilateral cleft lip and palate)	KOPSMCHBM_0026_01_CHILD009_R00209S8A2M0000P0000_C1_AV6UOS_A00985	105.2	13.40%	16,996	170	96%
68049	MOTHER9	Female	Negative	KOPSMCHBM_0027_01_MOTHER09_R00209S8A2M0001P0000_C1_AV6UOS_A00986	107	11.60%	16,440	164	93%
61779	CHILD10	Male	UCL/P (unilateral cleft lip and palate)	KOPSMCHBM_0028_01_CHILD010_R00210S8A2M0000P0000_C1_AV6UOS_A00987	116.2	11.90%	18,211	182	93%
61258	MOTHER10	Female	Negative	KOPSMCHBM_0029_01_MOTHER10_R00210S8A2M0001P0000_C1_AV6UOS_A00988	112.3	11.40%	17,089	171	92%
61259	FATHER10	Male	Negative	KOPSMCHBM_0030_01_FATHER10_R00210S8A2M0000P0001_C1_AV6UOS_A00989	105.4	13.90%	16,775	168	95%
61509	CHILD11	Male	UCL (unilateral cleft lip)	KOPSMCHBM_0031_01_CHILD011_R00211S8A2M0000P0000_C1_AV6UOS_A00990	94.3	11.70%	14,767	148	92%
61346	MOTHER11	Female	Negative	KOPSMCHBM_0032_01_MOTHER11_R00211S8A2M0001P0000_C1_AV6UOS_A00991	88.4	13.40%	14,839	148	92%
61345	FATHER11	Male	Negative	KOPSMCHBM_0033_01_FATHER11_R00211S8A2M0000P0001_C1_AV6UOS_A00992	109.3	10.10%	16,763	168	94%
61322	CHILD12	Male	UCL/P (unilateral cleft lip and palate)	KOPSMCHBM_0034_01_CHILD012_R00212S8A2M0000P0000_C1_AV6UOS_A00993	106.2	12.00%	16,286	163	95%
63688	FATHER12	Male	Negative	KOPSMCHBM_0035_01_FATHER12_R00212S8A2M0000P0001_C1_AV6UOS_A00994	102.2	12.10%	16,083	161	95%
63647	MOTHER12	Female	Negative	KOPSMCHBM_0036_01_MOTHER12_R00212S8A2M0001P0000_C1_AV6UOS_A00995	119	12.70%	18,055	181	96%
61337	CHILD13	Female	UCL (unilateral cleft lip)	KOPSMCHBM_0037_01_CHILD013_R00213S8A2M0000P0000_C1_AV6UOS_A00996	100.9	13.40%	15,663	157	96%
61279	MOTHER13	Female	Negative	KOPSMCHBM_0038_01_MOTHER13_R00213S8A2M0001P0000_C1_AV6UOS_A00997	109.5	13.60%	16,719	167	94%
61299	FATHER13	Male	Negative	KOPSMCHBM_0039_01_FATHER13_R00213S8A2M0000P0001_C1_AV6UOS_A00998	121.2	13.30%	18,484	185	95%
61113	CHILD14	Female	UCL (unilateral cleft lip)	KOPSMCHBM_0040_01_CHILD014_R00214S8A2M0000P0000_C1_AV6UOS_A00999	89.8	10.80%	14,745	147	89%
63515	MOTHER14	Female	Negative	KOPSMCHBM_0041_01_MOTHER14_R00214S8A2M0001P0000_C1_AV6UOS_A01000	104.7	12.10%	15,991	160	94%
63485	FATHER14	Male	Negative	KOPSMCHBM_0042_01_FATHER14_R00214S8A2M0000P0001_C1_AV6UOS_A01001	99.6	11.60%	15,136	151	94%
63486	FATHER15	Male	Negative	KOPSMCHBM_0043_01_FATHER15_R00215S8A2M0000P0001_C1_AV6UOS_A01002	102.1	12.00%	15,553	156	94%
61115	CHILD15	Female	BCL/P (bilateral cleft lip and palate)	KOPSMCHBM_0044_01_CHILD015_R00215S8A2M0000P0000_C1_AV6UOS_A01003	122	13.70%	18,551	186	97%
63487	MOTHER15	Female	Negative	KOPSMCHBM_0045_01_MOTHER15_R00215S8A2M0001P0000_C1_AV6UOS_A01004	114.6	12.60%	17,450	175	95%
61595	CHILD16	Female	UCL/P (unilateral cleft lip and palate)	KOPSMCHBM_0046_01_CHILD016_R00216S8A2M0000P0000_C1_AV6UOS_A01005	80.2	12.30%	12,899	129	92%
61482	MOTHER16	Female	Negative	KOPSMCHBM_0047_01_MOTHER16_R00216S8A2M0001P0000_C1_AV6UOS_A01006	112.7	13.50%	17,806	178	95%
61483	FATHER16	Male	Negative	KOPSMCHBM_0048_01_FATHER16_R00216S8A2M0000P0001_C1_AV6UOS_A01007	119.9	13.60%	18,226	182	96%
61762	CHILD17	Female	UCL/P (unilateral cleft lip and palate)	KOPSMCHBM_0049_01_CHILD017_R00217S8A2M0000P0000_C1_AV6UOS_A01008	118.6	12.30%	18,215	182	95%
61225	FATHER17	Male	Negative	KOPSMCHBM_0050_01_FATHER17_R00217S8A2M0001P0000_C1_AV6UOS_A01009	100	11.20%	15,333	153	94%
63663	MOTHER17	Female	Negative	KOPSMCHBM_0051_01_MOTHER17_R00217S8A2M0000P0001_C1_AV6UOS_A01010	107	11.50%	16,300	163	94%
61332	CHILD18	Female	UCL (unilateral cleft lip)	KOPSMCHBM_0052_01_CHILD018_R00218S8A2M0000P0000_C1_AV6UOS_A01011	128	17.80%	19,635	196	98%
61298	MOTHER18	Female	Negative	KOPSMCHBM_0053_01_MOTHER18_R00218S8A2M0001P0000_C1_AV6UOS_A01012	117.9	12.90%	18,132	181	95%
61295	FATHER18	Male	Negative	KOPSMCHBM_0054_01_FATHER18_R00218S8A2M0000P0001_C1_AV6UOS_A01013	106.9	12.70%	16,376	164	94%
63925	CHILD19	Female	UCL/P (unilateral cleft lip and palate)	KOPSMCHBM_0055_01_CHILD019_R00219S8A2M0000P0000_C1_AV6UOS_A01014	114.7	13.50%	17,940	179	97%
67973	MOTHER19	Female	Negative	KOPSMCHBM_0056_01_MOTHER19_R00219S8A2M0001P0000_C1_AV6UOS_A01015	110	12.00%	16,956	170	94%
68047	FATHER19	Male	Negative	KOPSMCHBM_0057_01_FATHER19_R00219S8A2M0000P0001_C1_AV6UOS_A01016	95.8	11.40%	14,438	144	94%
61581	CHILD20	Female	UCL (unilateral cleft lip)	KOPSMCHBM_0058_01_CHILD020_R00220S8A2M0000P0000_C1_AV6UOS_A01017	122.9	12.70%	18,732	187	96%
61381	MOTHER20	Female	Negative	KOPSMCHBM_0059_01_MOTHER20_R00220S8A2M0001P0000_C1_AV6UOS_A01018	102.4	11.50%	15,741	157	92%
61370	FATHER20	Male	Negative	KOPSMCHBM_0060_01_FATHER20_R00220S8A2M0000P0001_C1_AV6UOS_A01019	106.9	11.70%	16,367	164	95%
67446	CHILD21	Male	UCL/P (unilateral cleft lip and palate)	KOPSMCHBM_0061_01_CHILD021_R00221S8A2M0000P0000_C1_AV6UOS_A01020	117.9	12.40%	18,586	186	94%
63423	FATHER21	Male	Negative	KOPSMCHBM_0062_01_FATHER21_R00221S8A2M0001P0000_C1_AV6UOS_A01021	107	12.60%	16,180	162	94%
68097	MOTHER21	Female	Negative	KOPSMCHBM_0063_01_MOTHER21_R00221S8A2M0000P0001_C1_AV6UOS_A01022	114.7	13.10%	17,435	174	96%
61584	CHILD22	Male	UCL/P (unilateral cleft lip and palate)	KOPSMCHBM_0064_01_CHILD022_R00222S8A2M0000P0000_C1_AV6UOS_A01023	120.1	13.30%	18,421	184	96%
61378	MOTHER22	Female	Negative	KOPSMCHBM_0065_01_MOTHER22_R00222S8A2M0001P0000_C1_AV6UOS_A01024	122.1	12.60%	18,807	188	96%
61368	FATHER22	Male	Negative	KOPSMCHBM_0066_01_FATHER22_R00222S8A2M0000P0001_C1_AV6UOS_A01025	81.9	10.80%	12,712	127	90%
61481	MOTHER23	Female	Negative	KOPSMCHBM_0067_01_MOTHER23_R00223S8A2M0001P0000_C1_AV6UOS_A01026	104.9	11.60%	16,738	167	94%
61484	FATHER23	Male	Negative	KOPSMCHBM_0068_01_FATHER23_R00223S8A2M0000P0001_C1_AV6UOS_A01027	96.5	11.00%	15,309	153	93%
61593	CHILD23	Male	UCL (unilateral cleft lip)	KOPSMCHBM_0069_01_CHILD023_R00223S8A2M0000P0000_C1_AV6UOS_A01028	102.6	11.70%	15,897	159	95%
61343	MOTHER24	Female	Negative	KOPSMCHBM_0070_01_MOTHER24_R00224S8A2M0001P0000_C1_AV6UOS_A01029	113.6	13.40%	17,482	175	95%
61344	FATHER24	Male	Negative	KOPSMCHBM_0071_01_FATHER24_R00224S8A2M0000P0001_C1_AV6UOS_A01030	108.9	13.30%	17,022	170	95%
61512	CHILD24	Male	UCL/P (unilateral cleft lip and palate)	KOPSMCHBM_0072_01_CHILD024_R00224S8A2M0000P0000_C1_AV6UOS_A01031	99.3	12.40%	15,188	152	93%
61552	CHILD25	Female	BCL/P (bilateral cleft lip and palate)	KOPSMCHBM_0073_01_CHILD025_R00225S8A2M0000P0000_C1_AV6UOS_A01032	110	13.80%	16,937	169	94%
61266	FATHER25	Male	Negative	KOPSMCHBM_0074_01_FATHER25_R00225S8A2M0000P0001_C1_AV6UOS_A01033	86.3	11.40%	13,991	140	92%
61265	MOTHER25	Female	Negative	KOPSMCHBM_0075_01_MOTHER25_R00225S8A2M0001P0000_C1_AV6UOS_A01034	108.1	13.50%	16,661	167	96%
61590	CHILD27	Male	UCL/P (unilateral cleft lip and/or palate)	KOPSMCHBM_0079_01_CHILD027_R00227S8A2M0000P0000_C1_AV6UOS_A01038	110.9	12.60%	16,888	169	93%
61379	FATHER27	Male	Negative	KOPSMCHBM_0080_01_FATHER27_R00227S8A2M0000P0001_C1_AV6UOS_A01039	97.9	12.30%	15,126	151	92%
61347	MOTHER27	Female	Negative	KOPSMCHBM_0081_01_MOTHER27_R00227S8A2M0001P0000_C1_AV6UOS_A01040	101.2	11.20%	15,232	152	93%

Supplementary Table S2. Seqnificant frequency of homozygouse Asian-ancestrial haplotype by genome-wide ancestry analysis.

Chromosome	Start Position	End Position	proportion children homozygous Asian	proportion parents homozygous Asian	ChiSquare	p value
2	174,296,170	176,045,832	0.577	0.308	5.246	0.022
2	176,045,832	177,497,614	0.577	0.308	5.246	0.022
2	177,497,614	178,481,818	0.538	0.308	3.900	0.048
2	180,383,498	180,846,743	0.462	0.231	4.333	0.037
4	76,503,197	78,669,454	0.077	0.269	3.931	0.047
6	0	1,613,686	0.115	0.327	4.068	0.044
7	44,250,826	45,298,577	0.077	0.269	3.931	0.047
8	140,083,663	141,831,989	0.423	0.192	4.692	0.030
8	141,831,989	142,154,291	0.423	0.212	3.830	0.050
8	142,154,291	142,491,697	0.462	0.231	4.333	0.037
11	123,454,978	124,566,418	0.077	0.269	3.931	0.047
11	124,566,418	125,003,004	0.077	0.288	4.551	0.033
11	125,003,004	125,496,956	0.077	0.308	5.200	0.023
11	125,496,956	125,848,261	0.115	0.327	4.068	0.044
11	126,880,301	129,351,006	0.115	0.346	4.692	0.030
13	0	19,726,056	0.692	0.423	5.029	0.025
13	19,726,056	20,398,669	0.692	0.442	4.345	0.037
13	22,505,002	22,854,227	0.654	0.404	4.336	0.037
14	89,088,388	89,867,978	0.115	0.327	4.068	0.044
14	94,008,753	94,833,033	0.154	0.385	4.333	0.037
14	94,833,033	95,014,912	0.154	0.404	4.974	0.026
14	95,014,912	95,556,747	0.154	0.404	4.974	0.026
15	84,408,634	86,697,596	0.038	0.212	3.989	0.046
18	0	905,537	0.000	0.135	3.845	0.050

Supplementary Table S3. Total copy number variant aberrations and QC across samples.

Sample_ID	Family_ID	Sequencing_ID	Quality	Total CN aberrations
61780	CHILD1	KOPSMCHBM_0003_01_CHILD001_R00201S8A2M0000P0000_C1_AV6UOS_A00962	0.024	104
63450	MOTHER1	KOPSMCHBM_0001_01_MOTHER01_R00201S8A2M0001P0000_C1_AV6UOS_A00960	0.031	99
61290	FATHER1	KOPSMCHBM_0002_01_FATHER01_R00201S8A2M0000P0001_C1_AV6UOS_A00961	0.018	88
61600	CHILD2	KOPSMCHBM_0004_01_CHILD002_R00202S8A2M0000P0000_C1_AV6UOS_A00963	0.016	95
61494	MOTHER2	KOPSMCHBM_0006_01_MOTHER02_R00202S8A2M0001P0000_C1_AV6UOS_A00965	0.016	94
61495	FATHER2	KOPSMCHBM_0005_01_FATHER02_R00202S8A2M0000P0001_C1_AV6UOS_A00964	0.019	80
61772	CHILD3	KOPSMCHBM_0007_01_CHILD003_R00203S8A2M0000P0000_C1_AV6UOS_A00966	0.020	105
61374	MOTHER3	KOPSMCHBM_0008_01_MOTHER03_R00203S8A2M0001P0000_C1_AV6UOS_A00967	0.017	89
61373	FATHER3	KOPSMCHBM_0009_01_FATHER03_R00203S8A2M0000P0001_C1_AV6UOS_A00968	0.027	91
64005	CHILD4	KOPSMCHBM_0010_01_CHILD004_R00204S8A2M0000P0000_C1_AV6UOS_A00969	0.015	99
63702	MOTHER4	KOPSMCHBM_0012_01_MOTHER04_R00204S8A2M0001P0000_C1_AV6UOS_A00971	0.018	95
63536	FATHER4	KOPSMCHBM_0011_01_FATHER04_R00204S8A2M0000P0001_C1_AV6UOS_A00970	0.012	107
81700	CHILD5	KOPSMCHBM_0013_01_CHILD005_R00205S8A2M0000P0000_C1_AV6UOS_A00972	0.022	91
68046	MOTHER5	KOPSMCHBM_0015_01_MOTHER05_R00205S8A2M0001P0000_C1_AV6UOS_A00974	0.017	101
68019	FATHER5	KOPSMCHBM_0014_01_FATHER05_R00205S8A2M0000P0001_C1_AV6UOS_A00973	0.016	92
82207	CHILD6	KOPSMCHBM_0016_01_CHILD006_R00206S8A2M0000P0000_C1_AV6UOS_A00975	0.018	83
63713	MOTHER6	KOPSMCHBM_0017_01_MOTHER06_R00206S8A2M0001P0000_C1_AV6UOS_A00976	0.029	91
66533	FATHER6	KOPSMCHBM_0018_01_FATHER06_R00206S8A2M0000P0001_C1_AV6UOS_A00977	0.022	94
61516	CHILD7	KOPSMCHBM_0019_01_CHILD007_R00207S8A2M0000P0000_C1_AV6UOS_A00978	0.017	72
61210	MOTHER7	KOPSMCHBM_0020_01_MOTHER07_R00207S8A2M0001P0000_C1_AV6UOS_A00979	0.012	75
61213	FATHER7	KOPSMCHBM_0021_01_FATHER07_R00207S8A2M0000P0001_C1_AV6UOS_A00980	0.015	88
81695	CHILD8	KOPSMCHBM_0023_01_CHILD008_R00208S8A2M0000P0000_C1_AV6UOS_A00982	0.017	110
63629	MOTHER8	KOPSMCHBM_0022_01_MOTHER08_R00208S8A2M0001P0000_C1_AV6UOS_A00981	0.014	92
63715	FATHER8	KOPSMCHBM_0024_01_FATHER08_R00208S8A2M0000P0001_C1_AV6UOS_A00983	0.015	87
81716	CHILD9	KOPSMCHBM_0026_01_CHILD009_R00209S8A2M0000P0000_C1_AV6UOS_A00985	0.018	101
68049	MOTHER9	KOPSMCHBM_0027_01_MOTHER09_R00209S8A2M0001P0000_C1_AV6UOS_A00986	0.018	88
68037	FATHER9	KOPSMCHBM_0025_01_FATHER09_R00209S8A2M0000P0001_C1_AV6UOS_A00984	0.024	97
61779	CHILD10	KOPSMCHBM_0028_01_CHILD010_R00210S8A2M0000P0000_C1_AV6UOS_A00987	0.023	109
61258	MOTHER10	KOPSMCHBM_0029_01_MOTHER10_R00210S8A2M0001P0000_C1_AV6UOS_A00988	0.020	104
61259	FATHER10	KOPSMCHBM_0030_01_FATHER10_R00210S8A2M0000P0001_C1_AV6UOS_A00989	0.015	93
61509	CHILD11	KOPSMCHBM_0031_01_CHILD011_R00211S8A2M0000P0000_C1_AV6UOS_A00990	0.023	105
61346	MOTHER11	KOPSMCHBM_0032_01_MOTHER11_R00211S8A2M0001P0000_C1_AV6UOS_A00991	0.019	98
61345	FATHER11	KOPSMCHBM_0033_01_FATHER11_R00211S8A2M0000P0001_C1_AV6UOS_A00992	0.017	81
61322	CHILD12	KOPSMCHBM_0034_01_CHILD012_R00212S8A2M0000P0000_C1_AV6UOS_A00993	0.033	111
63688	FATHER12	KOPSMCHBM_0035_01_FATHER12_R00212S8A2M0000P0001_C1_AV6UOS_A00994	0.016	92
63647	MOTHER12	KOPSMCHBM_0036_01_MOTHER12_R00212S8A2M0001P0000_C1_AV6UOS_A00995	0.014	99
61337	CHILD13	KOPSMCHBM_0037_01_CHILD013_R00213S8A2M0000P0000_C1_AV6UOS_A00996	0.014	92
61279	MOTHER13	KOPSMCHBM_0038_01_MOTHER13_R00213S8A2M0001P0000_C1_AV6UOS_A00997	0.019	100
61299	FATHER13	KOPSMCHBM_0039_01_FATHER13_R00213S8A2M0000P0001_C1_AV6UOS_A00998	0.015	87
61113	CHILD14	KOPSMCHBM_0040_01_CHILD014_R00214S8A2M0000P0000_C1_AV6UOS_A00999	0.027	117
63515	MOTHER14	KOPSMCHBM_0041_01_MOTHER14_R00214S8A2M0001P0000_C1_AV6UOS_A01000	0.017	88
63485	FATHER14	KOPSMCHBM_0042_01_FATHER14_R00214S8A2M0000P0001_C1_AV6UOS_A01001	0.016	102
61115	CHILD15	KOPSMCHBM_0044_01_CHILD015_R00215S8A2M0000P0000_C1_AV6UOS_A01003	0.013	92
63487	MOTHER15	KOPSMCHBM_0045_01_MOTHER15_R00215S8A2M0001P0000_C1_AV6UOS_A01004	0.012	88
63486	FATHER15	KOPSMCHBM_0043_01_FATHER15_R00215S8A2M0000P0001_C1_AV6UOS_A01002	0.015	93
61595	CHILD16	KOPSMCHBM_0046_01_CHILD016_R00216S8A2M0000P0000_C1_AV6UOS_A01005	0.039	122
61482	MOTHER16	KOPSMCHBM_0047_01_MOTHER16_R00216S8A2M0001P0000_C1_AV6UOS_A01006	0.020	91
61483	FATHER16	KOPSMCHBM_0048_01_FATHER16_R00216S8A2M0000P0001_C1_AV6UOS_A01007	0.012	81
61762	CHILD17	KOPSMCHBM_0049_01_CHILD017_R00217S8A2M0000P0000_C1_AV6UOS_A01008	0.016	84
63663	MOTHER17	KOPSMCHBM_0051_01_MOTHER17_R00217S8A2M0001P0000_C1_AV6UOS_A01010	0.024	83
61225	FATHER17	KOPSMCHBM_0050_01_FATHER17_R00217S8A2M0001P0000_C1_AV6UOS_A01009	0.016	91
61332	CHILD18	KOPSMCHBM_0052_01_CHILD018_R00218S8A2M0000P0000_C1_AV6UOS_A01011	0.053	211
61298	MOTHER18	KOPSMCHBM_0053_01_MOTHER18_R00218S8A2M0001P0000_C1_AV6UOS_A01012	0.014	91
61295	FATHER18	KOPSMCHBM_0054_01_FATHER18_R00218S8A2M0000P0001_C1_AV6UOS_A01013	0.015	84
63925	CHILD19	KOPSMCHBM_0055_01_CHILD019_R00219S8A2M0000P0000_C1_AV6UOS_A01014	0.027	124
67973	MOTHER19	KOPSMCHBM_0056_01_MOTHER19_R00219S8A2M0001P0000_C1_AV6UOS_A01015	0.019	92
68047	FATHER19	KOPSMCHBM_0057_01_FATHER19_R00219S8A2M0000P0001_C1_AV6UOS_A01016	0.016	78
61581	CHILD20	KOPSMCHBM_0058_01_CHILD020_R00220S8A2M0000P0000_C1_AV6UOS_A01017	0.027	217
61381	MOTHER20	KOPSMCHBM_0059_01_MOTHER20_R00220S8A2M0001P0000_C1_AV6UOS_A01018	0.020	86
61370	FATHER20	KOPSMCHBM_0060_01_FATHER20_R00220S8A2M0000P0001_C1_AV6UOS_A01019	0.013	86
67446	CHILD21	KOPSMCHBM_0061_01_CHILD021_R00221S8A2M0000P0000_C1_AV6UOS_A01020	0.017	84
63423	FATHER21	KOPSMCHBM_0062_01_FATHER21_R00221S8A2M0001P0000_C1_AV6UOS_A01021	0.016	94
68097	MOTHER21	KOPSMCHBM_0063_01_MOTHER21_R00221S8A2M0000P0001_C1_AV6UOS_A01022	0.012	82
61584	CHILD22	KOPSMCHBM_0064_01_CHILD022_R00222S8A2M0000P0000_C1_AV6UOS_A01023	0.020	130
61378	MOTHER22	KOPSMCHBM_0065_01_MOTHER22_R00222S8A2M0001P0000_C1_AV6UOS_A01024	0.013	103
61368	FATHER22	KOPSMCHBM_0066_01_FATHER22_R00222S8A2M0000P0001_C1_AV6UOS_A01025	0.023	99
61593	CHILD23	KOPSMCHBM_0069_01_CHILD023_R00223S8A2M0000P0000_C1_AV6UOS_A01028	0.015	99
61481	MOTHER23	KOPSMCHBM_0067_01_MOTHER23_R00223S8A2M0001P0000_C1_AV6UOS_A01026	0.019	104
61484	FATHER23	KOPSMCHBM_0068_01_FATHER23_R00223S8A2M0000P0001_C1_AV6UOS_A01027	0.019	90
61512	CHILD24	KOPSMCHBM_0072_01_CHILD024_R00224S8A2M0000P0000_C1_AV6UOS_A01031	0.020	93
61343	MOTHER24	KOPSMCHBM_0070_01_MOTHER24_R00224S8A2M0001P0000_C1_AV6UOS_A01029	0.014	112
61344	FATHER24	KOPSMCHBM_0071_01_FATHER24_R00224S8A2M0000P0001_C1_AV6UOS_A01030	0.017	96
61552	CHILD25	KOPSMCHBM_0073_01_CHILD025_R00225S8A2M0000P0000_C1_AV6UOS_A01032	0.023	91
61265	MOTHER25	KOPSMCHBM_0075_01_MOTHER25_R00225S8A2M0001P0000_C1_AV6UOS_A01034	0.013	97
61266	FATHER25	KOPSMCHBM_0074_01_FATHER25_R00225S8A2M0000P0001_C1_AV6UOS_A01033	0.020	90
61590	CHILD27	KOPSMCHBM_0079_01_CHILD027_R00227S8A2M0000P0000_C1_AV6UOS_A01038	0.021	99
61347	MOTHER27	KOPSMCHBM_0081_01_MOTHER27_R00227S8A2M0001P0000_C1_AV6UOS_A01040	0.019	72
61379	FATHER27	KOPSMCHBM_0080_01_FATHER27_R00227S8A2M0000P0001_C1_AV6UOS_A01039	0.020	100

Supplementary Table S4. Focal copy number anlaysis in proband by GISTIC.

Region	Type	Q-Bound	G-Score	% of CNV Overlap	Is the copy number variant present in control present (Yes,No)?
chr1:152,187,775-152,188,275	CN Gain	8.10E-14	7.877191992	100	Yes
chr1:16,975,339-16,976,011	CN Gain	3.15E-10	5.375248132	100	Yes
chr3:195,510,921-195,512,221	CN Gain	8.10E-14	7.09958873	100	Yes
chr4:9,251,025-9,251,125	CN Gain	1.14E-09	5.094695732	100	Yes
chr5:21,481,722-21,483,422	CN Gain	8.10E-14	8.134686667	100	Yes
chr5:69,791,928-69,807,672	CN Gain	8.10E-14	7.766861252	100	Yes
chr5:768,947-796,041	CN Gain	2.69E-13	6.384846956	100	Yes
chr7:143,956,579-143,964,056	CN Gain	2.04E-10	5.461925667	100	Yes
chr8:7,581,375-7,620,612	CN Gain	8.10E-14	11.23369021	100	Yes
chr8:86,568,199-86,748,132	CN Gain	2.20E-10	5.440901408	100	Yes
chr8:7,879,217-7,879,844	CN Gain	1.90E-09	5.000535715	100	Yes
chr12:40,882,817-40,883,617	CN Gain	8.10E-14	9.952177374	100	Yes
chr12:11,527,143-11,546,116	CN Gain	1.74E-10	5.498513639	100	Yes
chr14:20,164,041-20,181,466	CN Gain	4.27E-11	5.721079677	100	Yes
chr15:102,293,960-102,294,560	CN Gain	8.10E-14	6.644038281	100	Yes
chr17:44,409,257-44,623,677	CN Gain	3.76E-13	6.316752911	100	Yes
chr18:44,555,453-44,555,753	CN Gain	8.10E-14	6.897743382	100	Yes
chr19:40,392,682-40,399,410	CN Gain	1.29E-09	5.062358141	100	Yes
chr22:20,372,718-20,397,138	CN Gain	8.10E-14	10.15842919	100	Yes
chr1:152,188,275-152,188,575	CN Loss	1.17E-06	13.10994023	100	Yes
chr1:25,599,191-25,643,377	CN Loss	8.39E-04	8.699069747	100	Yes
chr1:152,278,682-152,278,982	CN Loss	2.66E-03	6.432879817	100	Yes
chr3:195,513,221-195,513,321	CN Loss	3.01E-05	10.6147625	100	Yes
chr4:9,250,425-9,250,525	CN Loss	1.82E-03	6.66012746	100	Yes
chr5:180,365,675-180,430,813	CN Loss	7.64E-05	10.31412697	100	Yes
chr5:140,229,648-140,238,132	CN Loss	1.70E-03	6.690061927	100	Yes
chr5:21,482,322-21,483,422	CN Loss	6.37E-03	5.960769236	100	Yes
chr6:29,855,754-29,858,025	CN Loss	7.87E-09	20.37337758	100	Yes
chr8:11,994,746-11,999,696	CN Loss	2.23E-08	15.54705444	100	Yes
chr8:7,876,606-7,878,358	CN Loss	1.15E-03	8.36204952	100	Yes
chr10:124,361,424-124,376,751	CN Loss	1.15E-03	7.810786778	100	Yes
chr12:40,882,017-40,882,817	CN Loss	7.87E-09	20.78745687	100	Yes
chr12:40,874,770-40,875,966	CN Loss	6.82E-04	9.832211819	100	Yes
chr14:106,539,143-106,573,312	CN Loss	1.14E-04	10.12260732	100	Yes
chr14:105,415,210-105,415,810	CN Loss	4.51E-03	6.233637691	100	Yes
chr15:20,072,299-20,189,469	CN Loss	8.39E-04	8.780908505	100	Yes
chr15:25,331,366-25,334,079	CN Loss	1.27E-03	7.114733834	100	Yes
chr15:102,294,760-102,295,960	CN Loss	1.27E-03	7.112250015	100	Yes
chr16:16,345,772-16,360,952	CN Loss	8.39E-04	8.751494003	100	Yes
chr17:34,539,280-34,539,880	CN Loss	1.10E-06	13.42992342	100	Yes
chr18:44,552,446-44,555,353	CN Loss	9.03E-07	13.65889254	100	Yes
chr19:43,699,108-43,728,042	CN Loss	7.87E-09	18.68111245	100	Yes
chr19:41,354,195-41,356,311	CN Loss	6.82E-04	10.11539029	100	Yes
chr19:55,333,119-55,359,423	CN Loss	2.37E-03	6.539041266	100	Yes
chr22:24,342,956-24,384,346	CN Loss	7.87E-09	29.9881413	100	Yes
chr22:39,358,438-39,388,412	CN Loss	7.46E-04	9.737073481	100	Yes
chrX:49,267,446-49,324,846	CN Loss	4.51E-03	6.171917553	100	Yes

Supplementary Table S5. Copy number events only present in proband but not detected in the parents (control).

Position	Gene	CNVtype	Chromosome	start	end
chr20:62,329,994-62,339,365	ARFRP1	Loss	20	62,329,994	62,339,365
chr12:88,442,792-88,535,865	CEP290	Loss	12	88,442,792	88,535,865
chr1:192,127,591-192,154,945	RGS18	Loss	1	192,127,591	192,154,945
chr12:88,536,083-88,593,664	TMTC3	Loss	12	88,536,083	88,593,664

Supplementary Table S6. Additional details of variant description found in 26 Malagasy children with nCL/P.

FamilyID	Gene	Chromosome	Start	Stop	Reference	Genotype (Proband)	Genotype quality (infoGQ)	LRTscore	MutationAssessorScore	PPH2HumVarScore	PROVEANScore	Transcript	ensemblGene	HGVS (fullGNomen)	Protein (pNomen)
Child015	WNT5B	12	1742006	1742006	G	G|T	99	0	3.23	0.143	-4.47	NM_032642.3	ENSG00000111186	NC_000012.11:g.1742006G>T	p.R88L
Child015	GPC4	X	132440095	132440095	G	C|G	99	0	1.925	0.997	-3.84	NM_001448.3	ENSG00000076716	NC_000023.10:g.132440095G>C	p.A322G
Child005	MSX1	4	4861877	4861877	A	T|A	99	0.075	1.79	0.025	-1.5	NM_002448.3	ENSG00000163132	NC_000004.11:g.4861877A>T	p.E84V
Child006	MSX1	4	4861877	4861877	A	T|A	99	0.075	1.79	0.025	-1.5	NM_002448.3	ENSG00000163132	NC_000004.11:g.4861877A>T	p.E84V
Child009	MSX1	4	4861877	4861877	A	T|A	99	0.075	1.79	0.025	-1.5	NM_002448.3	ENSG00000163132	NC_000004.11:g.4861877A>T	p.E84V
Child023	MSX1	4	4861877	4861877	A	T|A	99	0.075	1.79	0.025	-1.5	NM_002448.3	ENSG00000163132	NC_000004.11:g.4861877A>T	p.E84V
Child005	SEPTIN9	17	75484846	75484846	G	A|G	99	0	2.27	0.995	-3.43	NM_006640.4	ENSG00000184640	NC_000017.10:g.75484846G>A	p.E370K
Child019	WDR11	10	122664299	122664299	A	G|A	99	0	2.215	0.824	-1.75	NM_018117.12	ENSG00000120008	NC_000010.10:g.122664299A>G	p.M1057V
Child004	PHGDH	1	120279876	120279876	C	T|T	99	0.055	1.18	0.617	-3.68	NM_006623.4	ENSG00000092621	NC_000001.10:g.120279876C>T	p.S311F
Child019	SKI	1	2160660	2160660	G	G|A	99	0	2.48	0.994	-4.51	NM_003036.4	ENSG00000157933	NM_003036.4:c.455G>A	p.R152H
